# Elevated Allochthony in Stream Food Webs as a Result of Longitudinal Cumulative Effects of Forest Management

**DOI:** 10.1007/s10021-021-00717-6

**Published:** 2021-10-22

**Authors:** Maitane Erdozain, Karen A. Kidd, Erik J. S. Emilson, Scott S. Capell, David P. Kreutzweiser, Michelle A. Gray

**Affiliations:** 1 Canadian Rivers Institute and Biology Department, University of New Brunswick, 100 Tucker Park Road, Saint John, New Brunswick E2L 4L5 Canada; 2 Department of Biology and School of Earth, Environment and Society, McMaster University, 1280 Main St. W., Hamilton, Ontario L8S 4K1 Canada; 3 Great Lakes Forestry Centre, Natural Resources Canada, Canadian Forest Service, 1219 Queen St. East, Sault Ste. Marie, Ontario P6A 2E5 Canada; 4 Canadian Rivers Institute and Faculty of Forestry and Environmental Management, University of New Brunswick, 28 Dineen Drive, Fredericton, New Brunswick E3B 5A3 Canada

**Keywords:** algae, autochthony, benthic macroinvertebrates, cumulative effect, forest harvesting, longitudinal trend, sculpin, stable isotope analysis

## Abstract

The river continuum concept (RCC) predicts a downstream shift in the reliance of aquatic consumers from terrestrial to aquatic carbon sources, but this concept has rarely been assessed with longitudinal studies. Similarly, there are no studies addressing how forestry related disturbances to the structure of headwater food webs manifest (accumulate/dissipate) downstream and/or whether forest management alters natural longitudinal trends predicted by the RCC. Using stable isotopes of carbon, nitrogen and hydrogen, we investigated how: 1) autochthony in macroinvertebrates and fish change from small streams to larger downstream sites within a basin with minimal forest management (New Brunswick, Canada); 2) longitudinal trends in autochthony and food web length compare among three basins with different forest management intensity [intensive (harvest and replanting), extensive (harvest only), minimal] to detect potential cumulative/dissipative effects; and 3) forest management intensity and other catchment variables are influencing food web dynamics. We showed that, as predicted, the reliance of some macroinvertebrate taxa (especially collector feeders) on algae increased from small streams to downstream waters in the minimally managed basin, but that autochthony in the smallest shaded stream was higher than expected based on the RCC (as high as 90% for some taxa). However, this longitudinal increase in autochthony was not observed within the extensively managed basin and was weaker within the intensively managed one, suggesting that forest management can alter food web dynamics along the river continuum. The dampening of downstream autochthony indicates that the increased allochthony observed in small streams in response to forest harvesting cumulates downstream through the river continuum.

**Supplementary Information:**

The online version contains supplementary material available at 10.1007/s10021-021-00717-6.

## Highlights

- Longitudinal increase in autochthony of stream consumers in reference basin
- Trends were weaker or nonexistent in basins with intensive and extensive forestry
- Evidence for downstream effects of forestry on stream food webs

## Introduction

The river continuum concept (RCC) predicts that stream food webs in forested catchments follow a longitudinal (upstream–downstream) gradient from reliance on terrestrially derived food sources (for example, leaf litter) in shaded headwaters, to aquatic sources (for example, algae) in mid-reaches, to particulate sources (seston) at the larger, downstream locations (Vannote and others 1980). Considering how deeply established this conceptual framework is in aquatic ecology, the scarcity of empirical evidence testing this prediction is surprising (Rosi-Marshall and others 2016). Multiple stream metabolism studies have documented longitudinal increases in gross primary production (GPP) and autotrophy (that is, GPP > respiration) in support of the RCC (for example, Bott and others 1985; McTammany and others 2003; Finlay 2011; Kaylor and others 2019). However, food webs can be decoupled from stream metabolism as exemplified by the heterotrophy paradox, where decomposers may contribute substantially to the heterotrophic state of a system via respiration of detritus but only minimally to animal production, which is mostly supported by autotrophy through the algae-grazer pathway (Thorp and Delong 2002). Therefore, food web studies that specifically assess the longitudinal changes in food use predicted by the RCC are warranted.

Although longitudinal increases in the autochthony of consumers have been reported in some systems (Finlay 2001; Rosi-Marshall and Wallace 2002), there is a growing body of food web research challenging some of the RCC predictions. For example, several studies have reported a considerable contribution of autochthonous food sources to food webs in small streams (for example, Lau and others 2009; Hayden and others 2016; Rosi-Marshall and others 2016; Erdozain and others 2019; Reis and others 2020) as well as in very large rivers (Delong and Thorp 2006; Thorp and Bowes 2017). These examples indicate that high-quality food sources such as algae (Guo and others 2016) contribute disproportionately more to animal production than would be predicted based on the limited algal production in small shaded streams or large turbid rivers (Marcarelli and others 2011). But with evidence also supporting the importance of terrestrial production as key basal resource for headwater food webs (for example, Wallace and others 1997; Reid and others 2008), debate over the relative importance and longitudinal patterns of the two sources continues (Brett and others 2017). It is also not clear the degree to which land use changes contribute to differences in the use of autochthonous food sources along a longitudinal gradient.

Anthropogenic catchment disturbances can alter stream food web dynamics by influencing resource availability and/or community structure, and this could have disproportionate effects downstream. Forest harvesting has been linked to increased algal production and autochthony in small streams due to an elevated delivery of nutrients and/or light availability (Rounick and others 1982; England and Rosemond 2004; Göthe and others 2009; but see Ishikawa and others 2016). But when riparian buffers are retained, as stipulated by management practices in most North American jurisdictions (Schilling 2009; McDermott and others 2010), a decreased reliance on algae has been documented in small streams, likely due to an elevated delivery of terrestrial materials such as sediments or dissolved organic carbon (DOC; Jonsson and others 2018; Erdozain and others 2019). These changes in the headwaters may have subsequent impacts downstream given the hydrological connectivity of fluvial systems. More specifically, the increased algal production in small streams resulting from canopy removal could either dissipate (Finlay 2011) or disproportionately affect productivity (Koenig and others 2019) downstream, potentially resulting in little or positive longitudinal changes in autochthony. In contrast, the accumulation of sediments and decrease in nutrients and autotrophic index of biofilms observed downstream in harvested catchments (Erdozain and others 2021a, 2021b) could lead to a longitudinal decrease in autochthony. Yet, to our knowledge, there are no studies addressing how forestry related disturbances to headwaters manifest (accumulate/dissipate) downstream and/or whether forest management alters natural longitudinal trends such as the increased autochthony predicted by the RCC. Considering the superior nutritional quality of algae, a decrease in its assimilation may result in a less efficient energy transfer to upper trophic levels (Brett and others 2017; Guo and others 2017) with potential implications for food web length (FWL) and macroinvertebrate/fish production (Finlay 2011; Kaylor and Warren 2017; Saunders and others 2018).

In this study, we investigated how food web structure (using C, H and N isotopes) changed along the river network within three basins differing in forest management intensity in New Brunswick (Canada) at a time of year when maximum autochthony would be likely. The objectives of the study were to assess how: 1) autochthony and FWL change from small streams to downstream waters within a basin with low forest management (minimal basin) to test the predictions of the RCC; 2) longitudinal trends in these measures compare among basins with different forest management intensity (intensive—includes replanting after harvesting, extensive—harvesting only, minimal; more details below) to detect potential cumulative or dissipative effects; and 3) forest management intensity and other catchment variables are influencing food web dynamics across this spatial scale. We predicted that 1) autochthony and FWL would increase downstream as predicted by the RCC, but that 2) the increase would be less pronounced in the intensively and extensively managed basins due to 3) autochthony and FWL being negatively affected by the elevated delivery of terrestrial materials resulting from increased forest management intensity.

## Methods

### Study Area

The study was conducted in three basins each established in areas of differing forest management in northern New Brunswick (NB, Canada) (Figure S1). The basin representing minimal management (NBR hereafter) is identified as a designated Watershed Protected Area by the Government of New Brunswick because it supplies municipal drinking water to the community and is therefore under stricter forest management guidelines (for example, wider riparian buffers, smaller cut blocks) (Government of New Brunswick 2020). The basin representing intensive forest management (NBI hereafter) is located in the Black Brook forestry district (privately owned and operated by J.D. Irving, Inc.). It is considered one of the most intensively managed forests in the country (Etheridge and others 2005) and implements artificial regeneration and various stand improvement interventions to maximize yield. The third basin represented a more extensive type of forest management (NBE) as forests are left to regenerate naturally after harvesting, resulting in less intervention and longer rotation cycles. It was not possible to find a reference basin of similar size to the other two that did not have any forest management. However, total disturbance (% of area with clearcut, partial harvest and replanting; more details in SI) from forestry was lowest in NBR (7.3% of the basin harvested in the 10 years prior to sampling) followed by NBE (12.7%) and NBI (23.0%). Site characteristics are shown in Table S1, and a more detailed characterization of the study areas is found in Erdozain and others (2021a).

Within each of the three basins, six stream sites were selected to represent an upstream–downstream gradient (stream orders 1–5). Because the contributing catchment area increases along this gradient, drainage area was used to quantitatively represent the upstream–downstream direction. Note that not all six sites were located along the same flowpath because of access constraints (Figure S1); however, we assumed that the same longitudinal processes were happening along different flowpaths within the same basin. The watershed of each site was delineated and characterized, yielding 18 sub-catchments that ranged in drainage area (0.7–233.5 km^2^), harvest intensity (0–23% of the catchment harvested in the 10 years prior to sampling), road density (1.30–3.58 km/km^2^), and forest structure (6–16 m average height) and composition (38–89% deciduous cover) (Table S1). This resulted in stream sites ranging in water chemistry (for example, 0.6–8.0 ppm DOC), dissolved organic matter quality (for example, 1.9–14.3 humification index), sediment deposition (for example, 0.1–1.9 g fine inorganic sediments) and water temperature (for example, 8.8–13.3 ºC in September) as explored in Erdozain and others (2021a) and available at https://doi.org/10.5683/SP2/B2URHU.

### Sample Collection

Food resources and macroinvertebrates were collected along a 100-m stream reach in September 2017 to match the timing of natural leaf fall. Due to the turnover time of consumer tissues, this timing likely reflected the summertime incorporation of food resources into food webs, that is, the time when maximum autochthony would be likely. Coarse particulate organic matter (CPOM) was sampled by collecting conditioned leaves from in-stream leaf accumulations, benthic fine particulate organic matter (FPOM) by suctioning the top centimeter of the substrate from depositional areas along the reach, and biofilm by scraping the surface of rocks and washing the slurry with stream water into bags (*n* = 3/site). Macroinvertebrates were collected by electroshocking and catching the drifting invertebrates with 363-µm mesh drift nets. Additionally, rocks and leaves were inspected to collect the invertebrates that are less likely to enter the drift (for example, *Glossosoma*). All the invertebrates were live-sorted to the lowest possible taxonomic level in the field, stored in bags partially filled with stream water and kept in the dark and on ice. All samples were frozen the same day until further analysis in the laboratory. Macroinvertebrates were not left to clear their guts overnight as our previous study found no effects of gut contents on the isotopic composition of similar taxa from the NBI watershed (Erdozain and others 2019). Slimy sculpin were collected about two weeks later from the same reach and transported to the laboratory in aerated stream water. After measuring length and body weight, fish were euthanized by cervical dislocation and frozen following the UNB Animal Care Committee approved protocol. No sculpin were caught at the smallest (the most upstream) site in NBI (NBI6). Water samples for H isotope analysis were collected along the reach, filtered through a 0.2-µm PES filter and kept cold and in the dark (3 subsamples per stream).

### Stable Isotope Analysis

CPOM samples were rinsed and oven-dried for 48 h at 60 °C and ground to a fine powder. Biofilm and FPOM samples were freeze-dried for 96 h. These powders were weighed into tin (3.00–3.20 mg for C and N isotopes) and silver (0.35–0.45 mg for H isotopes) capsules. Macroinvertebrates were identified to genus and classified according to their functional feeding group (FFG) using Merritt and others (2008). The taxa that were most widely represented across streams and that captured all five FFGs were selected for stable isotope analyses: *Glossosoma* (scraper), Heptageniidae (*Epeorus*, *Maccaffertium*, *Rhithrogena*; scraper), *Baetis* (collector-gatherer/scraper), *Ephemerella* (collector-gatherer), *Leuctra* (shredder), *Pteronarcys* (shredder), Hydropsychidae (*Parapsyche* and *Ceratopsyche*; collector-filterer), *Dolophilodes* (collector-filterer), Perlodidae (*Diura*, *Isogenoides*, *Isoperla*; predator) and *Sweltsa* (predator). Macroinvertebrate (*n* = 2–50/taxa/site; pooled whole bodies) and sculpin (*n* = 5–10/site; skinned muscle fillets from individuals) samples were freeze-dried for 48 h, ground to a fine powder and weighed into tin (0.40–1.20 mg for C and N isotopes) and silver (0.20–0.30 mg for H isotopes) capsules.

Carbon, nitrogen and hydrogen stable isotope ratios in food sources and consumers were measured at the Stable Isotope in Nature Laboratory (SINLab; Fredericton, New Brunswick, Canada). The analytical precision of internal standards was ± 0.06‰, 0.15‰ and 2.50‰, and duplicates within runs yielded average differences of 0.14‰, 0.12‰ and 4.3‰ (*n *= 28) for carbon, nitrogen and hydrogen, respectively. Water samples were analyzed for H isotope ratios at the Colorado Plateau Stable Isotope Laboratory (Flagstaff, Arizona, USA); the analytical precision of internal water standards was ± 0.17‰ on average. Stable isotope measurements are expressed as delta (*δ*) parts per thousand (‰) relative to the international standards Vienna PeeDee Belemnite for C, air for N, and Vienna Standard Mean Ocean Water for H, according to the equation:

$$\delta X = \left( {\frac{{R_{{{\text{sample}}}} }}{{R_{{{\text{standard}}}} }} - 1} \right) * 1000$$

where *X* is ^13^C, ^15^ N or ^2^H, and R is the corresponding ^13^C/^12^C, ^15^ N/^14^ N or ^2^H /^1^H ratios.

### Mixing Models

The relative contribution of food sources to the diets of macroinvertebrates and sculpin was estimated using a Bayesian 2-isotope (δ^13^C and δ^2^H), 2-source (algae—aquatic source/autochthony and CPOM—terrestrial source/allochthony) mixing model with MixSIAR (Stock and Semmens 2016) in R 3.6.1 (R Core Team 2019). Separate mixing models were performed for primary consumers (genus included as a fixed factor), predatory macroinvertebrates (genus included as a fixed factor) and sculpin within each site. Convergence of the models on the posterior distributions was determined before accepting the MixSIAR results with the diagnostic Gelman-Rubin and Geweke tests in MixSIAR.

After visualizing the data and prior to running these mixing models, several best practices were followed to ensure reliable and informative mixing model solutions (Philips and others 2014). Details on the specific adjustments made along with biplots are in Appendix S1, and include: 1) why biofilm samples were not a good representative of the aquatic food source and how algal isotope values were estimated to overcome this limitation; 2) the selection of only one (CPOM) out of two terrestrial food sources for mixing models; and 3) the selection of fractionation factors and adjustment for environmental water contributions. To complement and confirm the mixing model results, we also conducted simple regression analyses that did not rely on the assumptions that had to be made for mixing models (see below).

### Food web Length

The predictable increases in δ^15^N with each step in the food web have been used to calculate food web length in aquatic ecosystems following the equation: trophic position = λ + (*δ*^15^*N*_consumer_−δ^15^*N*_base_)/Δ_*n*_ [where *λ* is the trophic position of the organism used to estimate δ^15^N_base_]. Δ_n_ stands for the change in δ^15^N from source to consumer (that is, diet-tissue fractionation) and has been shown to range from 1.4 to 3.4‰ (Vander Zanden and Rasmussen 2001; Post 2002; McCutchan and others 2003, Bunn and others 2013). Considering the uncertainty around Δ_*n*_ estimates and how sensitive trophic level calculations are to Δ_*n*_ (Post 2002), herein we simply report the difference in δ^15^N between the top consumer (sculpin) and primary consumers as a proxy for FWL. Invertebrate primary consumers were used in place of algae as the baseline because consumers better integrate temporal variation in δ^15^N (Vander Zanden and Rasmussen 1999; Post 2002). Grazers and shredders had the lowest δ^15^N values, but because shredders were not represented in all 18 sites, three ubiquitous grazer taxa were selected (Heptageniidae, *Glossosoma* and *Baetis*) and the calculations done with the average δ^15^N values of the three taxa as a baseline.

### Catchment Explanatory Variables

Explanatory catchment variables describing the intensity of forest management (harvesting and roads), landscape characteristics (for example, drainage density, slope, wetness) and catchment forest condition (structure and composition) were calculated using provincial and J.D. Irving GIS data. Details on how these variables were calculated can be found in Appendix S1 as well as in Erdozain and others (2021a).

### Statistical Analysis

Differences in autochthony (that is, % algal contribution calculated using mixing models) among basins and taxa were assessed by running two-way analysis of variance (ANOVA) with Tukey’s post hoc tests. Relationships between autochthony and catchment explanatory variables were quantified by means of regression analyses that included basin type (intensive, extensive, minimal) and taxon as covariates to detect potential basin- and/or taxon-dependent relationships [Autochthony = Catchment variable x Basin type x Taxon]. Type II ANOVAs (*car* package) were used to test the significance of each variable and interaction term in the model. Regression models and potential interactions were visualized by plotting the relationship between autochthony and each explanatory variable for each basin and taxon separately. When significant interaction terms were detected, linear regressions were run separately for each basin and/or taxon to quantify and compare the response-explanatory variable associations among forest management types and/or taxa. A similar analysis to the one described for autochthony was performed for FWL estimates (δ^15^N range) but without taxon as a covariate in the models.

**Figure 1:**
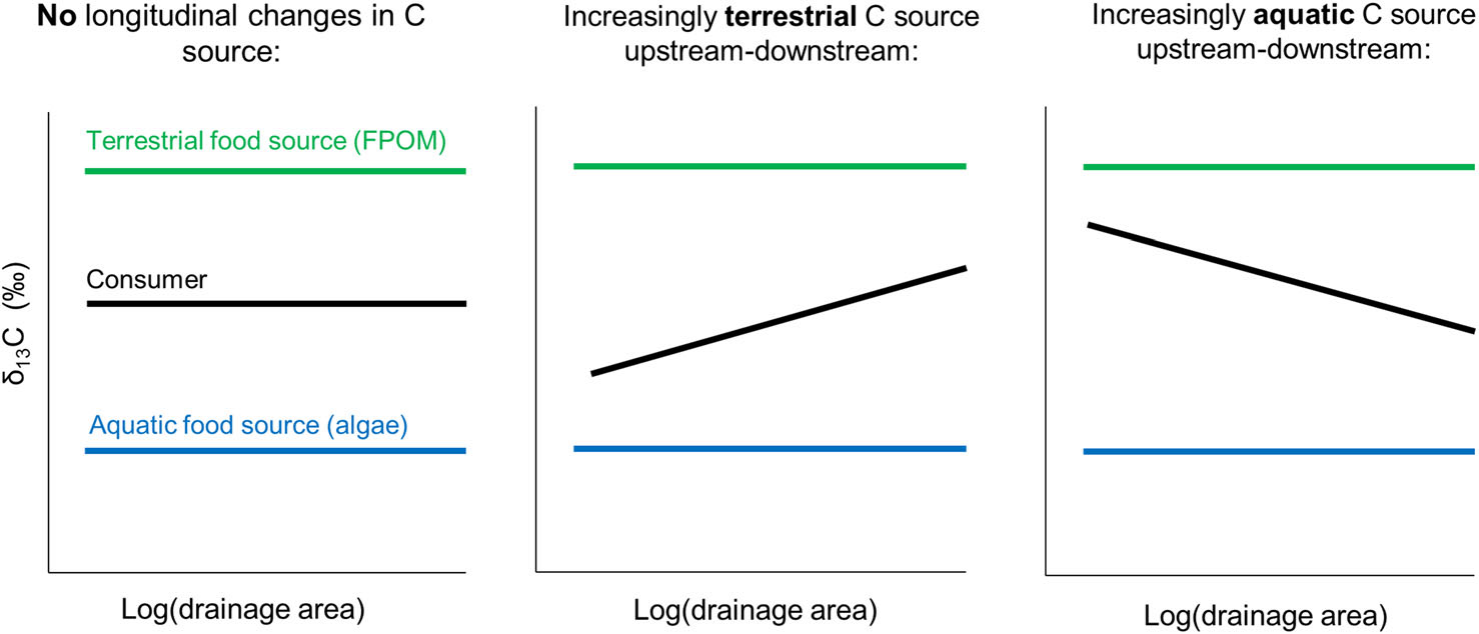
Theoretical framework used to assess how the diet of an aquatic consumer changes from upstream to downstream stream-sites using δ^13^C values.

The plots and regression model results with the natural logarithm of drainage area as explanatory catchment variable were used to examine whether: 1) autochthony or FWL showed longitudinal trends from small streams to downstream waters in the minimally managed basin to test the RCC, and 2) longitudinal trends varied among basins (that is, significant drainage area x basin interaction). For significant interactions, cumulative or dissipatory effects were inferred when forest management-related differences among basins increased or decreased longitudinally, respectively, in NBI or NBE relative to NBR. Additionally, a complementary approach was used to detect cumulative/dissipative effects that was free from the assumptions made for the mixing models (see *Mixing Models* section). Regression analyses were performed between raw isotope data and drainage area, and the slopes for consumers and food sources were compared (that is, consumer/food source x drainage area interaction tested). A significant interaction term between a particular consumer and a food source was interpreted as a longitudinal shift in diet, and the direction of the shift was determined by the sign of the slope (+ or− indicating an increasingly terrestrial or aquatic contribution, respectively) (Figure 1). The relatively constant δ^13^C and δ^2^H values for food sources among sites facilitated this approach. Regression analyses were done using CPOM as the reference food source to avoid the assumptions that had to be made when calculating algal values (see *Mixing Models* section), but calculated algal isotope values were also plotted to determine whether consumer slopes represented longitudinal changes in diet or longitudinal changes in algal δ^13^C. Alpha was set to 0.10 to compensate for the low sample size. Statistical analyses were performed in R 3.6.1 (R Core Team 2019).

## Results

### Testing the River Continuum Concept: Autochthony in a River Network with Minimal Forest Management

**Figure 2:**
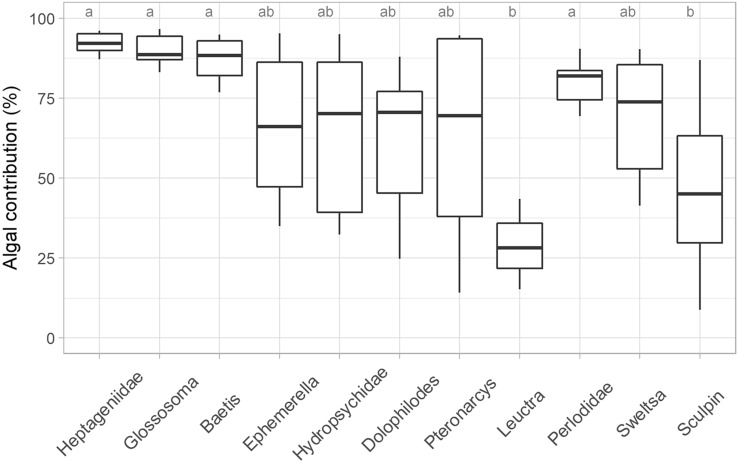
Boxplots showing the autochthony in different macroinvertebrate taxa and sculpin calculated based on C-H mixing models in the minimally managed basin (NBR). Letters represent significant (*p* ≤ 0.10) differences among taxa based on ANOVA and Tukey’s post hoc tests.

The mixing models indicated that autochthony differed significantly among taxa in NBR (*F*_10,49_ = 3.8, *p* < 0.001), with 9 of the 11 identified as having primarily autochthonous contributions (mean greater than 50%) at a time (September) when maximum autochthony would be likely (Figure 2, Table S2). Grazers (Heptageniidae and *Glossosoma*) and collector-gatherer *Baetis* showed the greatest autochthony, with average algal contributions of 92, 90 and 87%, respectively, and narrow ranges around the mean values. These taxa had significantly greater autochthony than sculpin (47%) and the shredder *Leuctra* (29%). Taxa with autochthony values between these highest and lowest estimates were the collector-gatherer *Ephemerella* (66% autochthony on average), two collector-filterers (Hydropsychidae—65% and *Dolophilodes*—61%), two predators (Perlodidae—80% and *Sweltsa*—69%) and the shredder *Pteronarcys* (62%).

**Figure 3:**
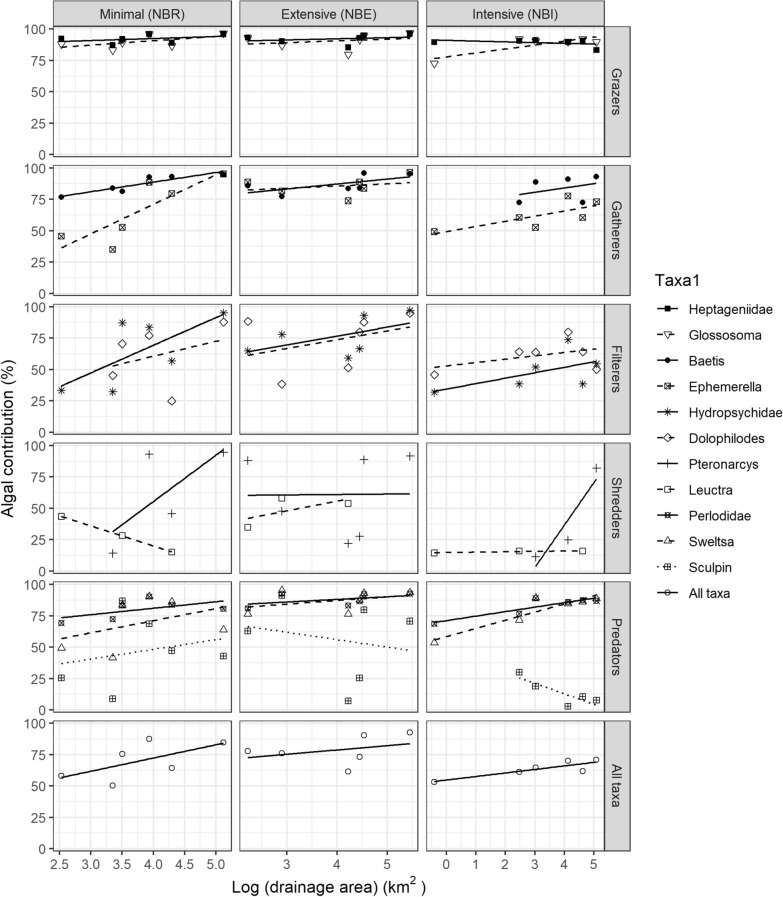
Linear relationship between autochthony (y-axis) in 8 invertebrate taxa and sculpin classified according to their functional feeding group (rows) and the logarithm of drainage area (x-axis) in three basins differing in forest management intensity (columns). Algal contribution was calculated using a Bayesian 2-isotope (δ^13^C and δ^2^H), 2-source (algae—autochthony and CPOM—allochthony) mixing model with six sites per basin.

Autochthony significantly increased with drainage area for taxa from NBR (Table 1). Within taxa, the longitudinal increase in autochthony was the clearest for *Baetis*, *Ephemerella* and Hydropsychidae, with the first two showing a significant increase (Table 1) and the latter two showing the steepest slopes (see first column in Figure 3).

**Table 1:** Results of Linear Regressions Between Autochthony in Different Macroinvertebrate Taxa or Sculpin and Drainage Area in the Minimally Managed Basin (NBR)

	*R*^*2*^	log(Area)	Taxon	log(Area)*Taxon
All taxa^1^	0.65	**0.002**	** < 0.001**	0.35
*Baetis*	0.84	**0.009**		
*Ephemerella*	0.70	**0.04**		
Heptageniidae	0.15	0.40		
*Glossosoma*	0.33	0.23		
Hydropsychidae	0.49	0.12		
*Dolophilodes*	0.11	0.59		
*Pteronarcys*	0.49	0.30		
Perlodidae	0.34	0.22		
*Sweltsa*	0.17	0.42		
Sculpin	0.06	0.64		

^1^Model tested: Algal contribution = Drainage area x Taxon.

The table shows the *R*^*2*^ and *p*-values from ANOVAs testing the significance of each fixed variable; *p values * ≤ 0.10 are bolded.

### Comparing Autochthony and FWL Among River Networks with Varying Forest Management Intensity

Mixing models indicated that overall mean autochthony significantly differed among basins (*F*_2,148_ = 10, *p* < 0.001) and was 14.2% greater at NBE than at NBI (*p* < 0.001) and 8% greater at NBE than at NBR (*p* = 0.03) when all taxa were pooled (Figure S5). Regarding longitudinal trends, the relationship between autochthony and drainage area was basin (management type) and taxon dependent (Table 2). Within basins and for all taxa combined, the longitudinal increase in autochthony observed within NBR was also observed within NBI but not within NBE. However, the downstream increase in autochthony was greater within NBR (autochthony was 27% greater downstream than upstream) than within NBI (18% greater) and NBE (15% greater). Within taxa, the interaction between drainage area and basin was significant for *Ephemerella*, with autochthony being 50% greater downstream than upstream within NBR, 24% greater within NBI and 8% greater within NBE (Figure 3). Autochthony in sculpin was 17% greater downstream than upstream within NBR, but 22% lower within NBI (that is, autochthony decreased longitudinally).

**Table 2:** Results of Linear Regressions Between Autochthony in Different Macroinvertebrate Taxa and Sculpin and Drainage Area in Three Basins Ranging in Forest Management Type

Within basin^1^			
	Drainage area	Taxon	Drainage area*Taxon
Intensive	** < 0.001**	** < 0.001**	** < 0.001**
Extensive	0.31	**0.006**	0.99
Minimal	**0.002**	** < 0.001**	0.35

Within taxa ^2^			
	Drainage area	Basin type	Drainage area*Basin type
*Baetis*	**0.02**	0.65	0.65
Heptageniidae	0.98	0.31	0.45
*Glossosoma*	**0.01**	0.99	0.75
*Ephemerella*	**0.01**	**0.02**	**0.02**
Hydropsychidae	**0.04**	0.14	0.22
*Dolophilodes*	0.30	0.62	0.80
*Pteronarcys*	0.26	0.62	0.33
*Leuctra*	0.88	**0.03**	0.13
Perlodidae	**0.005**	**0.10**	0.68
*Sweltsa*	**0.03**	0.11	0.72
Sculpin	**0.02**	0.65	0.65

^1^Within basin models: Algal contribution = Drainage area x Taxon.

^2^Within taxa models: Algal contribution = Drainage area x Basin type.

The table shows the *p*-values from ANOVAs testing the significance of each fixed variable in the within basin and within taxon models; *p values * ≤ 0.10 are bolded.

**Figure 4:**
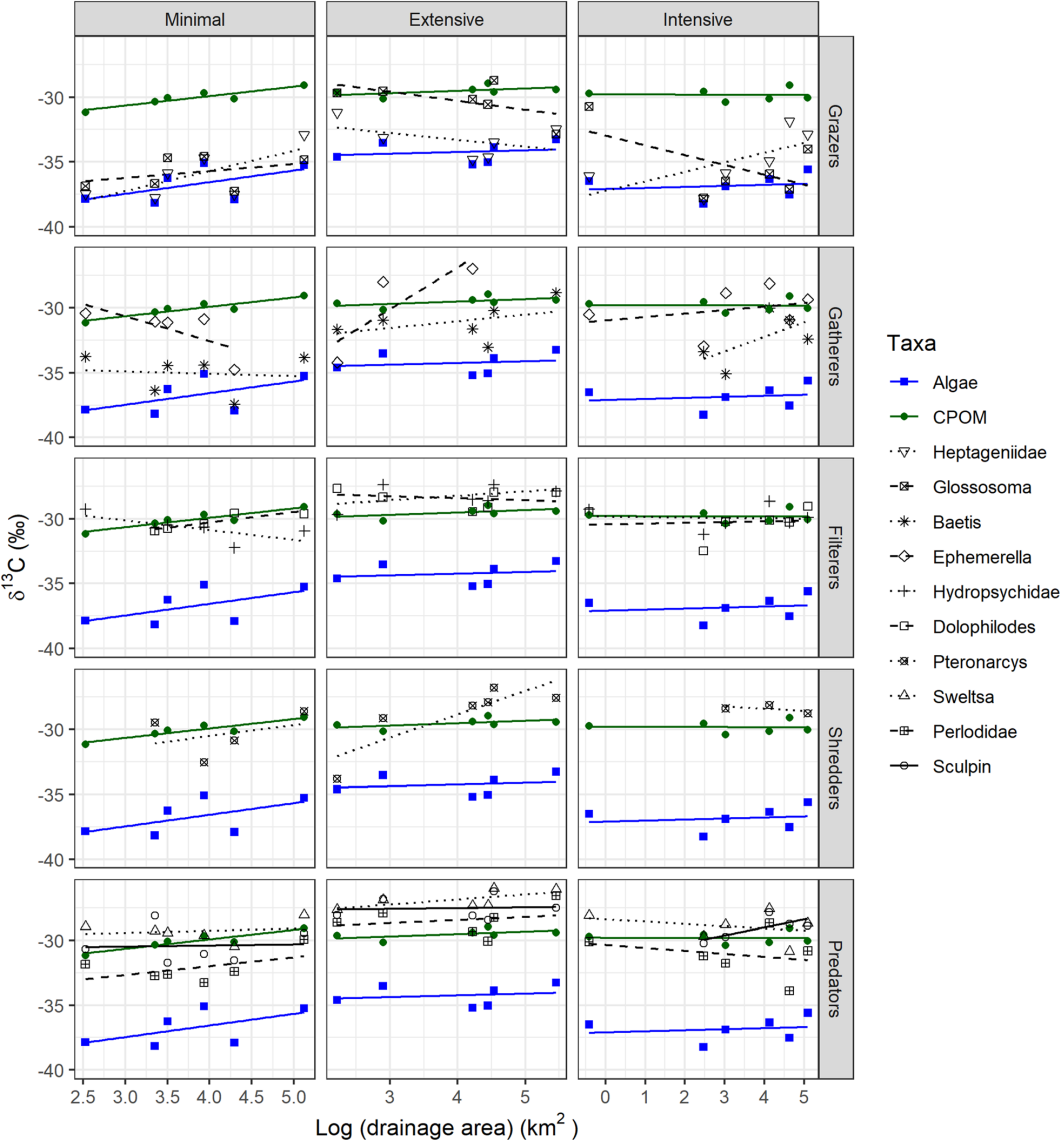
Linear relationship between δ^13^C and drainage area in stream consumers (black line, 8 invertebrate taxa and sculpin classified according to their functional feeding group (rows)) and food sources (terrestrial—green and aquatic—blue) in three basins differing in forest management intensity (columns). Six sites per basin were sampled.

When using raw δ^13^C values to assess longitudinal changes in food use, different spatial trends were also observed across basins (Figure 4, Table 3). The slopes of the relationships between δ^13^C values and drainage area differed between consumer and CPOM (that is, significant interaction) for several taxa, suggesting longitudinal changes in diet. At NBR, the interaction was significant for *Ephemerella* and Hydropsychidae: unlike δ^13^C in CPOM (and in calculated algae), consumer δ^13^C decreased longitudinally, suggesting a longitudinal decrease in terrestrial C reliance. At NBE, the opposite trend was observed for *Ephemerella* and *Pteronarcys*, as their δ^13^C values increased longitudinally, becoming more similar to those of CPOM and suggesting a longitudinal increase in terrestrial C reliance. At NBI, the δ^13^C values of Heptageniidae, *Baetis* and sculpin (significant interaction) became more positive along the gradient, indicating a greater reliance on terrestrial carbon downstream.

**Table 3:** Results of Linear Regressions Between δ^13^C in Terrestrial Food Sources (CPOM) and Stream Consumers (rows) and Drainage Area in Three Basins Ranging in Forest Management Intensity (columns)

	Intensive		Extensive		Minimal	
	*F*	*p*	*F*	*p*	*F*	*p*
*Baetis*	3.1	0.12	0.3	0.57	1.1	0.30
*Ephemerella*	0.4	0.54	7.7	**0.04**	9.6	**0.01**
Heptageniidae	3.1	0.12	1.8	0.21	1.1	0.32
*Glossosoma*	1.9	0.20	3.0	0.12	0.1	0.77
Hydropsychidae	0.01	0.92	0.2	0.69	12.9	**0.007**
*Dolophilodes*	0.04	0.85	1.1	0.32	0.1	0.77
*Pteronarcys*	0.2	0.65	7.7	**0.02**	0.01	0.90
*Sweltsa*	0.3	0.59	0.6	0.44	1.4	0.26
Perlodidae	0.2	0.62	0.009	0.93	0.006	0.94
Sculpin	4.0	**0.09**	0.1	0.75	0.6	0.44

The table shows the *F-* and *p*-values from ANOVAs testing the significance of the interaction term (that is, different slopes for CPOM and consumers); *p values * ≤ 0.10 are bolded.

**Figure 5:**
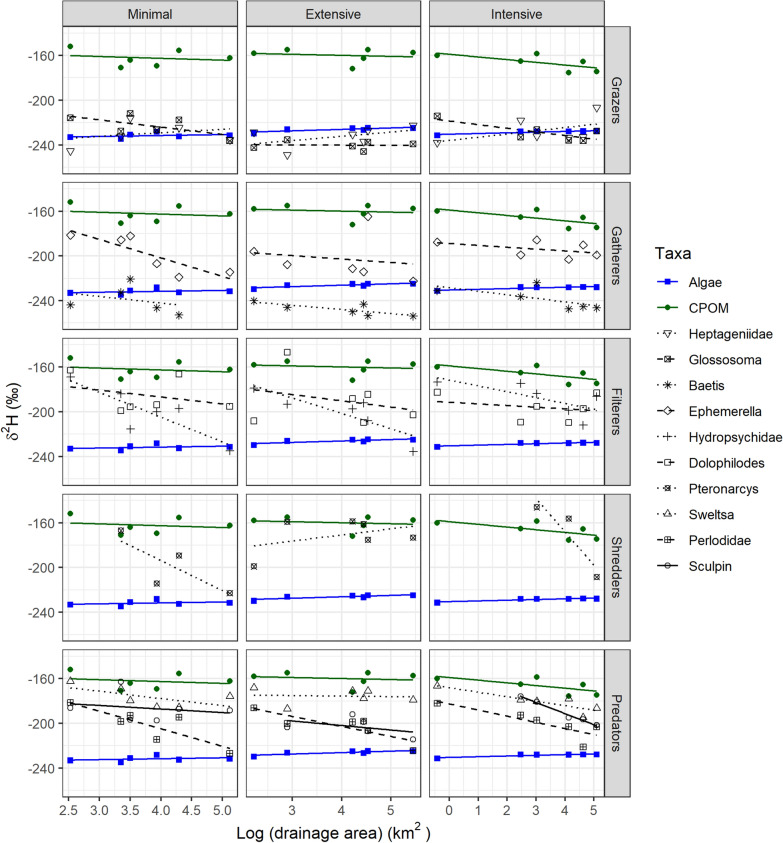
Linear relationship between δ^2^H in stream consumers (black line, 8 invertebrate taxa and sculpin classified according to their functional feeding group (rows)) and food sources (terrestrial—green and aquatic—blue) and drainage area in three basins differing in forest management intensity (columns). Six sites per basin were sampled.

The slope of the relationship between δ^2^H values and drainage area also differed between some consumers and food sources (Figure 5, Table S3). Within NBR, δ^2^H values in *Baetis*, *Ephemerella*, Hydropsychidae, *Pteronarcys* and Perlodidae became more negative downstream despite no change in food source δ^2^H, suggesting an increasingly aquatic diet. A similar trend was detected within NBE for Hydropsychidae and Perlodidae, and within NBI for *Pteronarcys*, Perlodidae, *Sweltsa* and sculpin.

**Figure 6:**
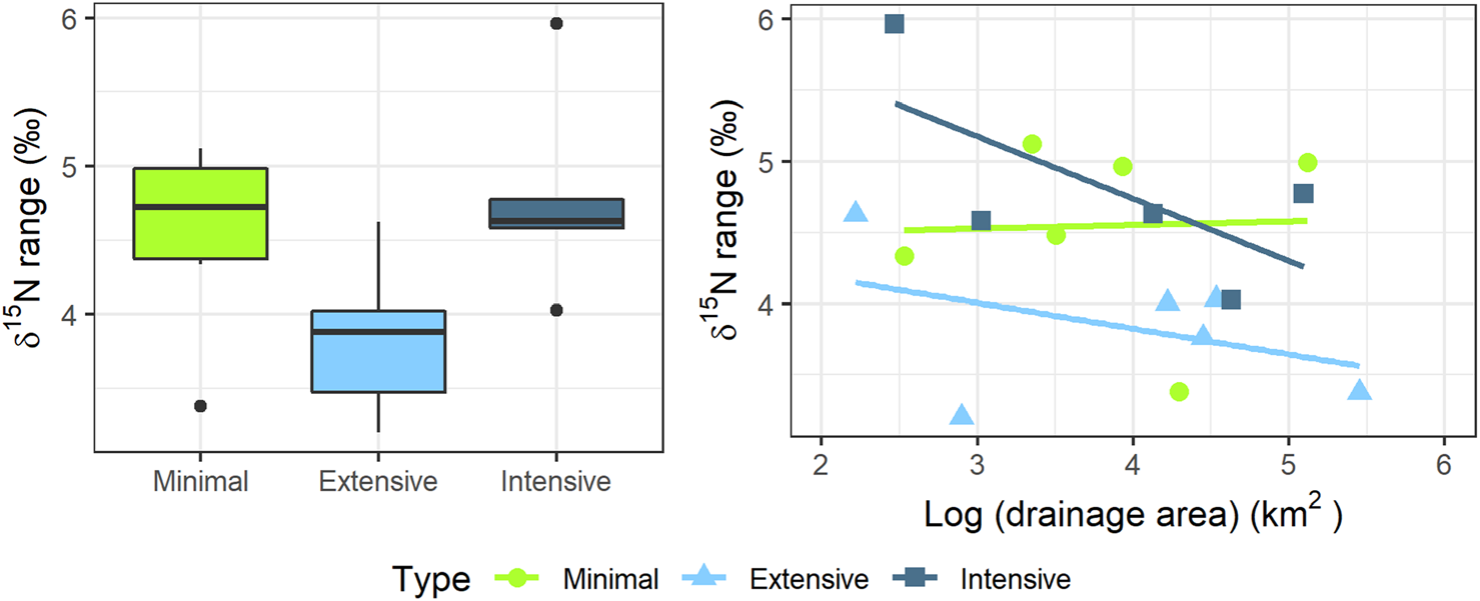
**a** Boxplot showing differences in the range in δ^15^N between sculpin and primary consumers (averaged *Baetis*, *Glossosoma* and Heptageniidae δ^15^N) and, **b** linear relationships between this range in δ^15^N and the logarithm of drainage area in three basins differing in forest management intensity.

FWL, calculated as the difference in δ^15^N between sculpin and primary consumers, was shorter within NBE than within NBI and NBR (*p* = 0.05) (Figure 6a). FWL was unrelated to drainage area; however, there was a nonsignificant negative trend at NBE and NBI, where FWL was shorter at the most downstream than upstream site, which was not observed in NBR (Figure 6b).

### The Effect of Forest Management and Other Catchment Variables

**Figure 7:**
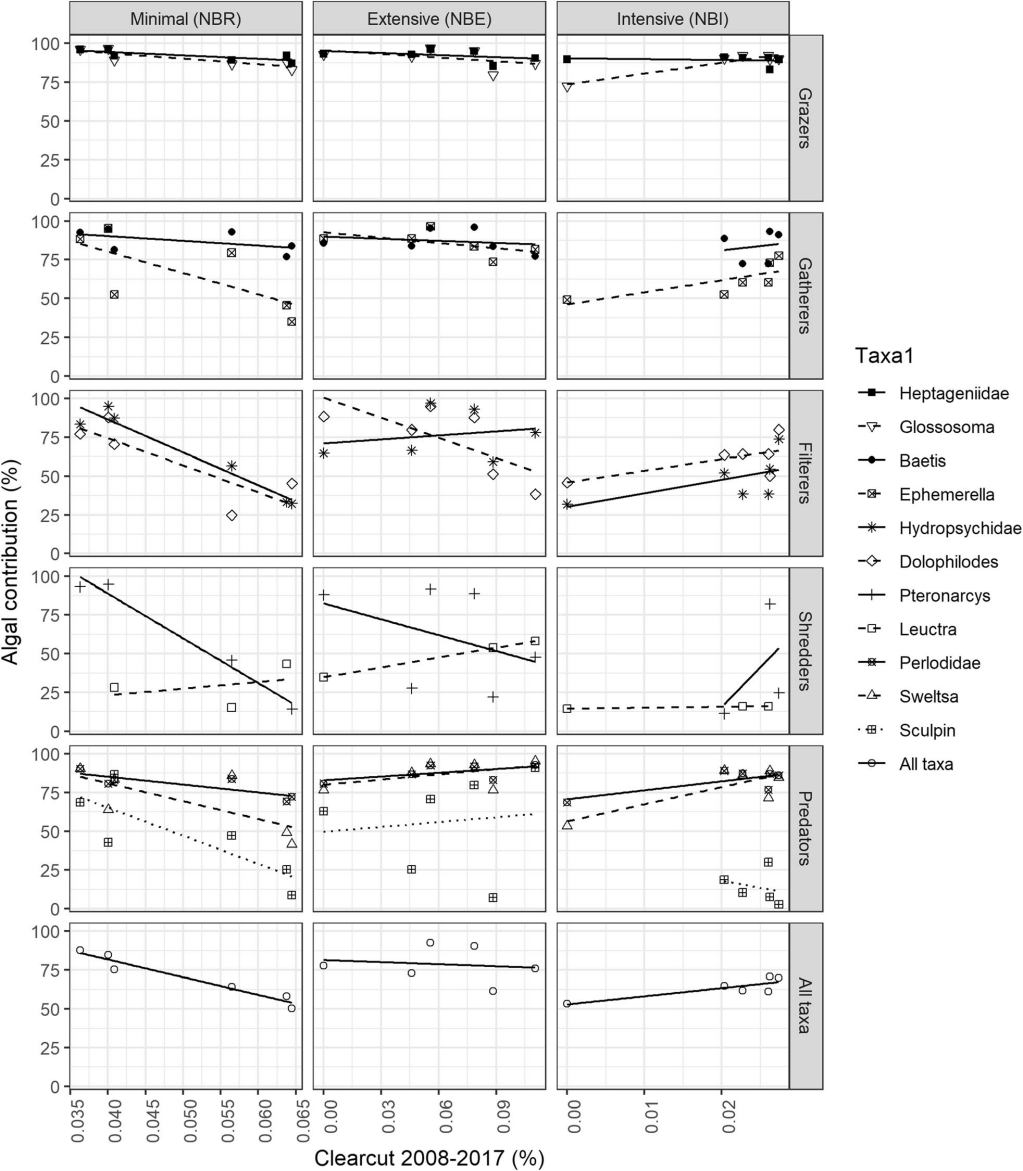
Linear relationship between autochthony (y-axis) in 8 invertebrate taxa and sculpin classified according to their functional feeding group (rows) and clearcut intensity between 2008 and 2017 (x-axis) in three basins differing in forest management intensity (columns). Six sites per basin were sampled.

Of the other catchment variables examined herein, autochthony of the consumers was significantly related to DTW < 0.1 m (that is, proportion of the catchment with depth-to-water values lower than 0.1 m, wet areas) and to % clearcut, but the latter relationship was basin and taxon dependent (Table 4). When all taxa were pooled, a significant decrease in autochthony with increasing % clearcut in NBR, a significant taxon-dependent increase in NBI and no relationship in NBE were found (Figure 7, Table S4). Heptageniidae was the only taxon that showed a consistent decrease in autochthony with clearcut across basins, but for several other taxa the relationship was basin dependent (Table S4). As examples, autochthony in *Dolophilodes* decreased with clearcut within NBR and NBE, but not within NBI, and autochthony in Perlodidae decreased with clearcut within NBR, but increased within NBI. Regarding DTW below 0.1, autochthony was significantly and positively related to this variable only in NBR (Figure S6).

**Table 4:** Results of Linear Regressions Between Autochthony in Invertebrate Taxa and Sculpin and Explanatory Catchment Variables (EV; Rows) in Three Basins Ranging in Forest Management Type (Minimal, Intensive and Extensive)

	EV	Type	Taxa	EV*Type	EV*Taxa	Type*Taxa	EV*Type*Taxa
Drainage area (log)	**0.03**	**0.09**	** < 0.001**	0.39	**0.10**	**0.005**	**0.07**
Crossing density	0.22	**0.04**	** < 0.001**	0.75	0.86	0.35	0.33
Road density	0.38	0.22	** < 0.001**	0.29	0.56	**0.006**	0.38
Clearcut	0.19	** < 0.001**	** < 0.001**	** < 0.001**	**0.02**	**0.003**	**0.01**
Total disturbance	0.36	**0.05**	** < 0.001**	0.34	0.63	0.41	0.13
Slope	0.50	0.15	** < 0.001**	0.79	0.88	0.39	0.99
DTW < 0.1 m	**0.01**	**0.002**	** < 0.001**	0.55	0.27	**0.002**	0.54
Forest height	0.93	0.29	** < 0.001**	0.41	**0.09**	0.37	0.39
Deciduous cover	0.73	**0.04**	** < 0.001**	0.24	0.83	**0.02**	0.10

The table shows the *p*-values from mixed model ANOVAs testing the significance of each fixed variable in the following model: Algal contribution = EV + Type + Taxon + EV*Type + EV*Taxon + Type*Taxon + EV*Type*Taxon + (1|Site); *p values * ≤ 0.10 are bolded.

FWL was also related to catchment variables, but these relationships also varied among basins. Within NBE, FWL significantly decreased with crossing density and total disturbance (Table S5). Within NBI, FWL significantly decreased with road density and increased with deciduous cover.

## Discussion

### Testing the RCC

The RCC predicts a downstream increase in autochthony in forested catchments (Vannote and others 1980), a prediction that we tested using the longitudinal trends within a basin with minimal forest management (NBR). In this basin, we found a significant increase in consumer autochthony with drainage area, consistent with the RCC, but this trend was taxon (and FFG) specific, as shown by others (Finlay 2001; Rosi-Marshall and Wallace 2002). Herein, the clearest and most consistent increases in autochthony were found in the collector-gatherers *Baetis* and *Ephemerella* and the collector-filterer Hydropsychidae. The wide range in their autochthony from upstream to downstream was notable (that is, 33% to 95% for Hydropsychidae, 45 to 95% for *Ephemerella*) and makes sense considering they are collectors and, thus, may better represent overall food availability compared to other more selective FFGs (for example, grazers). Our results match those of Finlay (2001), in which collectors and filterers showed the clearest longitudinal shifts from terrestrial to aquatic sources, but they contrast with studies that did not find longitudinal shifts in autochthony for these FFGs (Hayden and others 2016; Jonsson and others 2018). In the current study, the shredder *Leuctra* showed consistently low autochthony (~29%) along the gradient in our sites as described by others (Finlay 2001; Hayden and others 2016), but autochthony in the facultative shredder *Pteronarcys* increased from 14% in the smallest stream to 95% in the largest, supporting that this genus is able to adapt to changes in resource availability (Plague and others 1998; Rosi-Marshall and others 2016). In addition, in our study, there was also little evidence for longitudinal trends in the autochthony of predators along NBR: we detected an increase in autochthony for Perlodidae (only with *δ*^2^H) but no changes for *Sweltsa* or sculpin, which could be mostly feeding on a taxon that we did not collect (for example, Chironomidae; Arciszewski and others 2015). Overall, results show that predators were not selecting prey based on their degree of autochthony, as seen elsewhere (Lau and others 2014).

Autochthony levels of taxa in the small shaded streams within the basin with minimal harvesting were higher than would be expected based on the RCC. All taxa in the smallest NBR stream had autochthony values greater than 25% and as high as 92% for Heptageniidae, 88% for *Glossosoma* scrapers, and 69% for Perlodidae predators. Although these values should be considered with caution due to the assumptions that had to be made prior to running mixing models, they are consistent with other studies reporting high levels of autochthony in biota in small streams (for example, Lau and others 2009; Rosi-Marshall and others 2016; Hayden and others 2016; Erdozain and others 2019; Reis and others 2020) and suggest that high-quality food sources such as algae (Guo and others 2016) contribute more to animals than would be predicted based on the limited algae available. For this reason, both resource quantity and quality need to be considered to understand food web dynamics along fluvial systems (Marcarelli and others 2011). However, it is important to consider tissue turnover times and note that the timing of our sampling likely represented the maximum autochthonous resource incorporation; therefore, autochthony estimates would probably be lower later in the fall or winter months (Junker and Cross 2014). In addition, the distribution of FFGs herein did not match the RCC prediction of shredders dominating small, shaded streams: only 17.5% of the macroinvertebrates in the smallest stream were shredders, and some downstream sites had higher percentages (Erdozain and others 2021b), supporting claims that the distribution of FFGs along the river continuum is not a reliable indicator of resources consumed (Rosi-Marshall and others 2016). This discrepancy in longitudinal trends between autochthony and community composition may be linked to the dietary plasticity of some taxa along this continuum.

### The effect of forest management on food web structure

The longitudinal increase in autochthony for all consumers combined within the minimally managed basin was not found within the extensively managed basin and was weaker within the intensively managed one, suggesting that forest management affects some of the predictions made by the RCC (Vannote and others 1980). In fact, when comparing consumer δ^13^C to that of food sources along the gradient, some taxa showed a longitudinal decrease in aquatic C reliance within NBE (*Ephemerella* and *Pteronarcys*) and NBI (Heptageniidae and sculpin). These differences in the basins with greater forest management could result from a lower downstream availability of autochthonous food sources. Several abiotic and biotic indicators measured at these sites (for example, temperature, sediments, biofilm composition, DOC, nutrients; Erdozain and others 2021a, 2021b) support this hypothesis: 1) GPP (Saunders and others 2018; Kaylor and others 2019) and autochthony (Junker and Cross 2014) are controlled by water temperature, and this measure increased downstream in NBR, not at all in NBE and only weakly in NBI, mirroring the trends in autochthony reported herein; 2) the downstream increase in inorganic sediments was greatest at NBE and this could impair primary production through shading or scouring (Izagirre and others 2009; Jones and others 2012); 3) aerial diatom abundance in biofilms measured with a BenthoTorch (bbe moldaenke, Germany) decreased longitudinally at NBE; and 4) the longitudinal increase in DOC and decrease in phosphorus concentrations observed within NBI could have favored the heterotrophic over the autotrophic component of biofilms, and thus, limited the longitudinal increase in autochthony (Mindl and others 2005; Danger and others 2007). Collectively, results suggest that the downstream increase in autochthony is dampened within the basins with more harvesting. A loss of longitudinal trends related to catchment disturbance was also reported for primary production (Finlay 2011). Such a decrease in trophic diversity at the basin scale could have cascading ecological effects, and thus, additional examination of whether catchment disturbance diminishes trophic diversity along the river continuum, as well as its ecological implications, is recommended. Additionally, because the sampling was done at a time likely representing the maximum reliance on autochthonous food sources, studies investigating these questions during different seasons are recommended.

The negative effect of forest management on consumer autochthony was further supported by the negative relationship between autochthony and % clearcut detected herein. Increased clearcut intensity in these basins led to higher DOC concentrations of a more terrestrial origin as well as lower algal biomass on rocks (Erdozain and others 2021a, 2021b), explaining the negative effect of % clearcut on autochthony in these taxa. Our results contrast with studies reporting positive effects of harvesting on consumer autochthony at sites with no riparian buffers (Rounick and others 1982; England and Rosemond 2004; Göthe and others 2009) but concur with others that also detected negative effects of forestry on organism autochthony in the presence of buffers and consequent shading (Jonsson and others 2018; Erdozain and others 2019). Similarly, the negative relationship between consumer autochthony and % clearcut but not % partial harvest (strongly related to total disturbance herein) in the current study suggests that the complete removal of trees had a greater effect than partial harvest on food web dynamics, as was shown for DOC concentrations (Kreutzweiser and others 2008; Erdozain and others 2018) or sediment transport (Croke and Hairsine 2006). This could explain why the attenuation of longitudinal trends in autochthony was greater in NBE than NBI, as: 1) most of the harvest is partial rather than clearcut in NBI (Erdozain and others 2021a); and 2) the enhanced post-harvest regeneration practices (for example, planting, herbicides) applied in NBI speed up the recovery of the forest. Similarly, the very low % clearcut values and regeneration practices in NBI could explain why the negative effects of clearcut on consumer autochthony were not detected in this basin.

### C versus H Isotopes

Stable isotope ratios of carbon (δ^13^C) have been widely used as a tracer of the energy base supporting stream food webs. However, under certain conditions, aquatic and terrestrial food sources can overlap in their δ^13^C values (Finlay 2001), limiting the effectiveness of this tool in food web studies. Recently, stable isotope ratios of hydrogen (δ^2^H) have gained attention to complement δ^13^C due to the large difference in δ^2^H between aquatic and terrestrial food sources (Doucett and others 2007; Solomon and others 2009; Cole and others 2011). However, although both diet tracers would be expected to yield similar results, that was not the case in the present study nor in a review that found a surprising lack of correlation between allochthony estimates based on δ^13^C and δ^2^H (Brett and others 2018). Herein, autochthony estimates for NBE consumers were considerably lower based on δ^13^C than δ^2^H (see Figures 5 and 6), and we detected a longitudinal decrease in autochthony for some taxa in NBE and NBI using δ^13^C but an increase using δ^2^H. This lack of congruence has direct implications for conclusions drawn and suggests that one may be more reliable than the other in such studies. Since uncertainty remains around some key and influential assumptions for δ^2^H such as environmental water contributions, fractionation, routing or lipid extraction (Vander Zanden and others 2016; Newsome and others 2017; Brett and others 2018), we have put more weight on the δ^13^C results herein.

## Conclusions

We showed that the reliance of some macroinvertebrate taxa (especially collector feeders) on algae increased from small streams to downstream waters in the basin with minimal forest management as predicted by the river continuum concept (RCC). However, the basin with extensive forest management did not show the same longitudinal increase in consumer autochthony and the basin with intensive forest management showed a weaker increase, suggesting that forest management alters food web dynamics along the river continuum. This deviation from the RCC was mostly likely due to a greater delivery of terrestrial materials (DOC, sediments) as well as differences in the longitudinal trends in water temperatures observed at these more impacted sites. Finally, our results indicate that the increased allochthony observed in aquatic biota from small streams with forest harvesting also manifests downstream in a cumulative manner.

## Supplementary Information

Below is the link to the electronic supplementary material.

Supplementary file1 (DOCX 3073 kb)

## Data Availability

Data are available at https://doi.org/10.17632/5xdw5ysk5y.1.
