# Supplementary material for: Elevated Allochthony in Stream Food Webs as a Result of Longitudinal Cumulative Effects of Forest Management
Source: Ecosystems. 2021 Oct 22;25(6):1311–27. doi: 10.1007/s10021-021-00717-6 (PMC9519712; doi:10.1007/s10021-021-00717-6)
Supplement: Supplementary file 1 — Supplementary file1 (DOCX 3073 kb) [file 10021_2021_717_MOESM1_ESM.docx]

**Appendix S1**

Erdozain M, Kidd KA, Emilson EJS, Capell SS, Kreutzweiser DP, Gray MA. Elevated allochthony in stream food webs as a result of longitudinal cumulative effects of forest management.

# Study area and catchment variables

**Table S1:** UTM coordinates (19T zone) and catchment characteristics of the 18 sampling sites (DTW – mean depth-to-water).

| Stream-site | X | Y | Drainage area (km^2^) | Total disturbance (%, 2008-17) | Clearcut (%, 2008-17) | Crossing density (#/km) | Road density (km/km^2^) | Forest height (m) | Deciduous cover (%) | DTW (m) |
| --- | --- | --- | --- | --- | --- | --- | --- | --- | --- | --- |
| NBE1 | 47.36078 | -68.07194 | 233.5 | 12.7 | 5.6 | 0.45 | 2.41 | 13.2 | 63.9 | 22.9 |
| NBE2 | 47.41159 | -68.07556 | 85.3 | 16.7 | 4.6 | 0.56 | 2.69 | 10.6 | 71.7 | 27.1 |
| NBE3 | 47.43935 | -68.06444 | 9.2 | 0.0 | 0.0 | 0.08 | 3.58 | 16.0 | 78.4 | 27.1 |
| NBE4 | 47.36435 | -68.04194 | 93.2 | 11.2 | 7.9 | 0.34 | 2.17 | 14.2 | 56.6 | 18.6 |
| NBE5 | 47.39061 | -68.01389 | 68.0 | 13.1 | 8.8 | 0.31 | 2.31 | 13.8 | 57.7 | 18.7 |
| NBE6 | 47.41004 | -68.02500 | 18.1 | 14.6 | 11.0 | 0.48 | 2.73 | 14.5 | 76.2 | 22.1 |
| NBI1 | 47.43016 | -67.83639 | 163.0 | 23.0 | 2.6 | 0.78 | 2.43 | 6.5 | 68.8 | 25.2 |
| NBI2 | 47.45257 | -67.87028 | 20.6 | 18.8 | 2.0 | 0.47 | 2.13 | 7.7 | 71.0 | 23.5 |
| NBI3 | 47.48940 | -67.90139 | 11.8 | 20.5 | 2.6 | 0.61 | 1.91 | 6.9 | 80.7 | 21.9 |
| NBI4 | 47.46766 | -67.90750 | 102.5 | 21.2 | 2.3 | 0.92 | 2.49 | 6.5 | 71.5 | 26.1 |
| NBI5 | 47.49055 | -67.95722 | 62.0 | 20.3 | 2.7 | 0.83 | 2.33 | 6.4 | 73.1 | 25.4 |
| NBI6 | 47.55868 | -68.00972 | 0.7 | 6.4 | 0.0 | 0.00 | 1.30 | 6.1 | 89.0 | 22.9 |
| NBR1 | 47.94969 | -66.40167 | 167.5 | 7.3 | 4.0 | 0.35 | 1.99 | 13.9 | 49.4 | 17.8 |
| NBR2 | 47.86021 | -66.57194 | 33.2 | 5.5 | 4.1 | 0.36 | 1.95 | 15.1 | 41.5 | 16.3 |
| NBR3 | 47.91020 | -66.51417 | 51.0 | 11.5 | 3.6 | 0.37 | 2.14 | 13.0 | 52.3 | 11.9 |
| NBR4 | 47.86387 | -66.54222 | 73.2 | 6.7 | 5.6 | 0.36 | 2.00 | 15.0 | 46.1 | 17.5 |
| NBR5 | 47.85406 | -66.55833 | 28.5 | 7.4 | 6.4 | 0.38 | 2.04 | 14.8 | 47.8 | 16.4 |
| NBR6 | 47.81680 | -66.58472 | 12.5 | 6.4 | 6.4 | 0.17 | 1.70 | 13.8 | 38.4 | 13.0 |


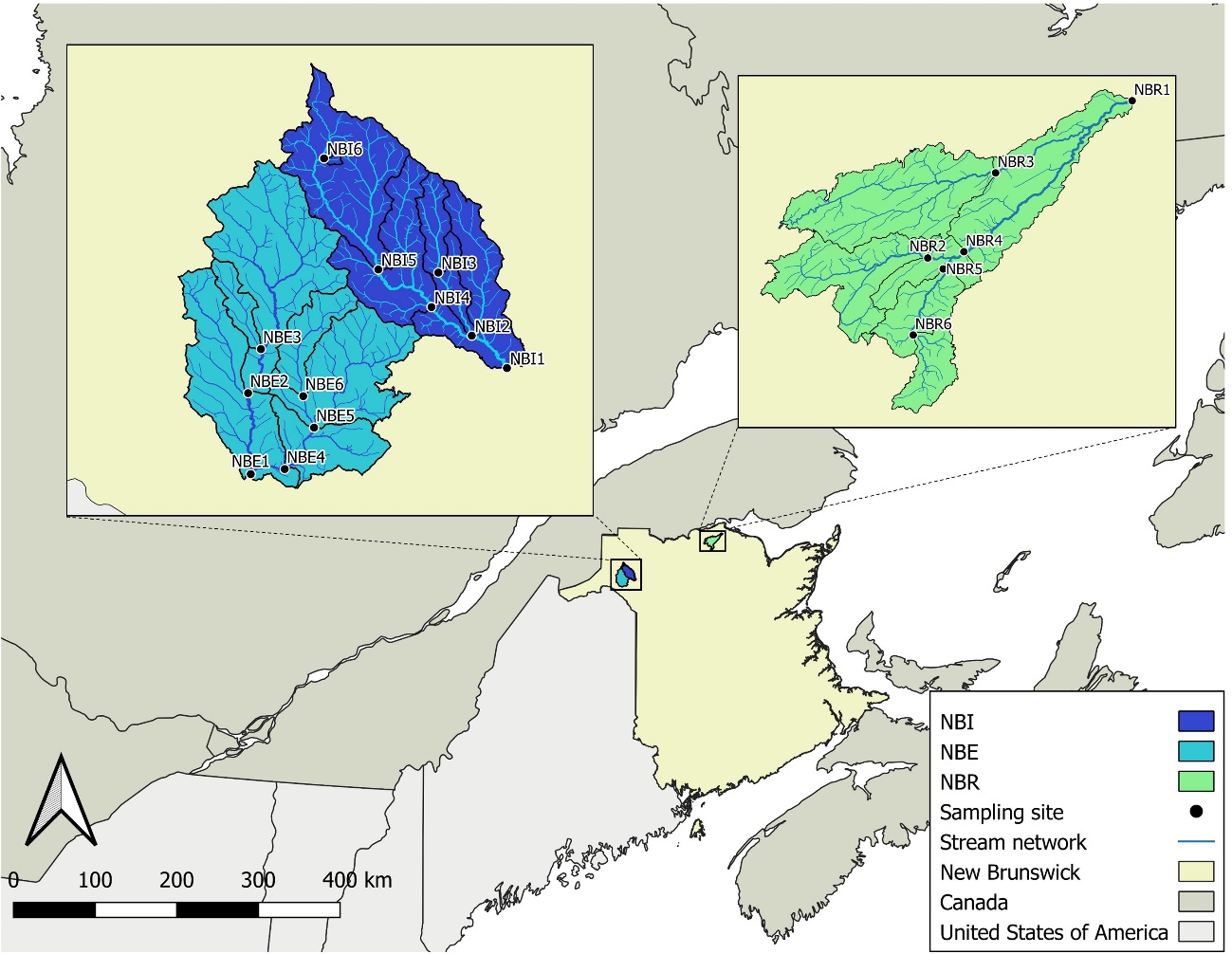
**Fig. S1:** Map showing the location of New Brunswick (Canada), the three study basins (NBE – extensively managed, NBI – intensively managed, NBR – reference) and the 18 stream sites and corresponding sub-catchments.

Explanatory variables for the catchments were classified into three categories: forest management (harvesting and roads), landscape characteristics, and forest condition (structure and composition). Harvest variables were calculated from the GIS information on stands harvested each year, available from the province (NBE, NRB) and J.D. Irving (NBI). In each catchment, the area harvested each year by different methods was calculated and divided by total area to calculate the percentage of the catchment harvested. Harvesting method was either clearcut, in which ~>80% of the trees are removed, or partial, in which ~35-50% of the trees are removed. Total management disturbance was the sum of % clearcut, % partial harvest and % artificial regeneration. These yearly values were then summarized into variables of the cumulative percentage of the catchment harvested by different methods in the last 5 and 10 years prior to sampling (e.g., 2008-2017 and 2009-2018 for 10 years). Road variables were calculated from the road shapefiles obtained from GeoNB and included road crossings (number of times that a road crosses a stream upstream from each sampling site), road crossing density (road crossings divided by stream length), road length (sum of all road lengths in the catchment) and road density (road length divided by the area of the study catchment) (Table S1).

Several landscape features that could potentially affect stream ecosystems were quantified from the 20-m provincial DEM and stream shapefile using Whitebox GAT (Lindsay 2016), and included catchment area, drainage density (stream length divided by catchment area), mean catchment slope, mean catchment elevation and catchment elevation range. To characterize catchment wetness, depth-to-water (DTW) values were calculated in ArcGIS as described by Murphy et al. (2011), and mean catchment DTW (Table S1) and % DTW <0.1, 0.1-1, 1-20 and >20 m (not shown) were calculated.

Forest condition variables were derived from the provincial and J.D. Irving forest resource inventories. Forest structure was quantified by calculating the average height, crown closure, vertical stand structure and developmental stage. Forest composition was characterized by calculating the relative area covered by each tree species in the overstory (>2 m height) and understory (<2 m) layers; these data were summarized by running a non-metric multidimensional scaling analysis and calculating deciduous vs. coniferous cover.

*Literature cited:*

Lindsay, J.B. 2016. Whitebox GAT: A case study in geomorphometric analysis. Computers & Geosciences 95:75-84.

Murphy, P.N.C., J. Ogilvie, F-R. Meng, B. White, J.S. Bhatti, and P.A. Arp. 2011. Modelling and Mapping Topographic Variations in Forest Soils at High Resolution: A Case Study. Ecological Modelling 222(14): 2314–32.

# Biplots and best practices for mixing models

After visualizing the data and prior to running these mixing models, several best practices were followed to ensure reliable and informative mixing model solutions (Philips et al. 2014). First, from the biplots it was clear that biofilm samples were not a good representative of the aquatic food source as some consumers (namely grazers) fell outside the mixing polygon defined by food sources (more negative δ^13^C and δ^2^H than biofilm) and biofilm samples overlapped with terrestrial food sources in some streams (Fig. S2-S4). As previously reported, this is because biofilms consist of a mixture of autotrophic (algae) and heterotrophic (bacteria, fungi) organisms and detritus but some consumers selectively ingesting/assimilating the autotrophic component (McNeely et al. 2007, Chessman et al. 2009, Rasmussen 2010). A potential solution for obtaining the isotopic value of primary producers is to use values of herbivores as proxies (Vander Zanden and Rasmussen 1999, Finlay 2001). We estimated algal δ^13^C from the primary consumer with the most negative δ^13^C values (after adjusting for fractionation, see below) in each stream, as this was interpreted to indicate that this taxon had the greatest algal assimilation. These taxa were either Heptageniidae or *Glossosoma* grazers depending on the stream, which are known to feed selectively on microscopic algae on biofilms (Oemke 1984, McNeely et al. 2006, Katano and Doi 2014). δ^2^H values of algae were estimated by subtracting 150 ± 25‰ from stream water δ^2^H (ranging between -74.5 and -84.7‰) based on studies showing that primary producers fractionate against ^2^H during photosynthesis, resulting in ~150‰ more negative values than environmental water (Hondula et al. 2014, Brett et al. 2018).

Second, as the two terrestrial food sources (CPOM and FPOM) had very similar δ^13^C and δ^2^H values, and as we were mostly interested in measuring allochthony *vs.* autochthony rather than in whether consumers were feeding on CPOM or FPOM, only one terrestrial food source (CPOM) was selected for mixing models. CPOM was considered to be a better representative of terrestrial food sources due to being less processed and less heterogeneous than FPOM (Wotton 2007).

Third, fractionation factors of 0.4 ± 1.20‰ for δ^13^C (McCutchan et al. 2003) and 0‰ for δ^2^H (Solomon et al. 2009) were assumed. Genera-specific enrichment factors were not available for the macroinvertebrates collected herein. As such, these average values were used although we recognize that this likely does not reflect true values across the different taxa included herein. Because a fraction of an organism’s ^2^H comes from dietary water rather than assimilated food, this was accounted for by incorporating in the mixing models the dietary water contribution (δ^2^H_WC_), which was calculated according to:

${\text{δ}^{\text{2}}\text{H}}_{\text{WC}}\text{= }{\text{δ}^{\text{2}}\text{H}}_{\text{cons}}\text{-(}{\text{δ}^{\text{2}}\text{H}}_{\text{cons}}\text{-}\text{ω}_{\text{tot}}\text{*}{\text{δ}^{\text{2}}\text{H}}_{\text{water}}\text{)/(1-}\text{ω}_{\text{tot}}\text{)}$ ,

where δ^2^H_water_ is the δ^2^H of stream water, and ω_tot_ is the total contribution of dietary water to consumer δ^2^H (δ^2^H_cons_), calculated according to:

$$\text{ω}_{\text{tot}}\text{=1-}\left( \text{1- ω} \right)^{\text{τ}}\text{ ,}$$

where ω is the per-trophic-level contribution of dietary water to consumers and τ is the trophic level (τ = 1 for primary consumers and 2 for predators). A ω of 0.20 ± 0.1 was assumed based on values from Solomon et al. (2009), Wang et al. (2009), Wilkinson et al. (2015) and Erdozain et al. (2019).

This third step is recognized to be one of the biggest sources of uncertainty -considering the variability in fractionation factors and environmental contribution estimates (Post 2002, Newsome et al. 2017, Brett et al. 2018) and how sensitive mixing models are to variation in these estimates (Bond and Diamond 2011, Brett et al. 2018). To complement and confirm the mixing model results, we conducted a simple analysis based on regressions that did not rely on the above-mentioned assumptions (see main article).

*Literature cited:*

Bond, A.L., and A.W. Diamond. 2011. Recent Bayesian Stable-Isotope Mixing Models Are Highly Sensitive to Variation in Discrimination Factors. Ecological Applications 21(4):1017–23.

Brett, MT, GW Holtgrieve, and DE Schindler. 2018. An Assessment of Assumptions and Uncertainty in Deuterium-Based Estimates of Terrestrial Subsidies to Aquatic Consumers. Ecology 99 (5):1073–88.

Chessman, B.C., D.P. Westhorpe, S.M. Mitrovic, and L. Hardwick. 2009. Trophic Linkages between Periphyton and Grazing Macroinvertebrates in Rivers with Different Levels of Catchment Development. Hydrobiologia 625(1):135–50.

Erdozain, M., K.A. Kidd, D.P. Kreutzweiser, and P.K. Sibley. 2019. Increased Reliance of Stream Macroinvertebrates on Terrestrial Food Sources Linked to Forest Management Intensity. Ecological Applications 29(4):e01889.

Finlay, J.C. 2001. Stable-Carbon-Isotope Ratios of River Biota: Implications for Energy Flow in Lotic Food Webs. Ecology 82(4):1052–64.

Hondula, K.L., M.L. Pace, J.J. Cole, and R.D. Batt. 2014. Hydrogen Isotope Discrimination in Aquatic Primary Producers: Implications for Aquatic Food Web Studies. Aquatic Sciences 76(2):217–29.

Katano, I., and H. Doi. 2014. Stream Grazers Determine Their Crawling Direction on the Basis of Chemical and Particulate Microalgal Cues. PeerJ 2:e503.

McCutchan, J.H., W.M. Lewis, C. Kendall, and C.C. McGrath. 2003. Variation in Trophic Shift for Stable Isotope Ratios of Carbon, Nitrogen, and Sulfur. Oikos102:378–90.

McNeely, C., J.C. Finlay, and M.E. Power. 2007. Grazer Traits, Competition, and Carbon Sources to a Headwater-Stream Food Web. Ecology 88(2):391–401.

McNeely, C., S.M. Clinton, and J.M. Erbe. 2006. Landscape Variation in C Sources of Scraping Primary Consumers in Streams. Journal of the North American Benthological Society 25(4):787–99.

Newsome, SD, N Wolf, CJ Bradley, and ML Fogel. 2017. Assimilation and Isotopic Discrimination of Hydrogen in Tilapia: Implications for Studying Animal Diet with Δ2H. Ecosphere 8(1).

Oemke, M.P. 1984. Interactions between a Stream Grazer and the Diatom Flora. In Proceedings of the Fourth International Symposium on Trichoptera. Clemson, South Carolina, 11-16 July 1983, edited by J.C. Morse, 291–99. Dordrecht, The Netherlands: Springer Netherlands.

Phillips, D.L., R. Inger, S. Bearhop, A.L. Jackson, J.W. Moore, A.C. Parnell, B.X. Semmens, and E.J. Ward. 2014. Best Practices for Use of Stable Isotope Mixing Models in Food-Web Studies. Canadian Journal of Zoology 835 (August): 823–35.

Post, D.M. 2002. Using Stable Isotopes to Estimate Trophic Position: Models, Methods, and Assumptions. Ecology 83(3):703–18.

Rasmussen, J.B. 2010. Estimating Terrestrial Contribution to Stream Invertebrates and Periphyton Using a Gradient-Based Mixing Model for D13C. Journal of Animal Ecology 79(2):393–402.

Solomon, C.T., J.J. Cole, R.R. Doucett, M.L. Pace, N.D. Preston, L.E. Smith, and B.C. Weidel. 2009. The Influence of Environmental Water on the Hydrogen Stable Isotope Ratio in Aquatic Consumers. *Oecologia* 161(2):313–24.

Vander Zanden, M.J., and J.B. Rasmussen. 1999. Primary Consumer δ13C and δ15N and the Trophic Position of Aquatic Consumers. Ecology 80(4):1395–1404.

Wang, Y.V., D.M. O’Brien, J. Jenson, D. Francis, and M.J. Wooller. 2009. The Influence of Diet and Water on the Stable Oxygen and Hydrogen Isotope Composition of Chironomidae (Diptera) with Paleoecological Implications. Oecologia 160 (2): 225–33.

Wilkinson, G.M., J.J. Cole, and M.L. Pace. 2015. Deuterium as a Food Source Tracer: Sensitivity to Environmental Water, Lipid Content, and Hydrogen Exchange. Limnology and Oceanography: Methods 13 (5): 213–23.

Wotton, R.S. 2007. Do benthic biologists pay enough attention to aggregates formed in the water column of streams and rivers? Journal of the North American Benthological Society 26(1): 1-11.


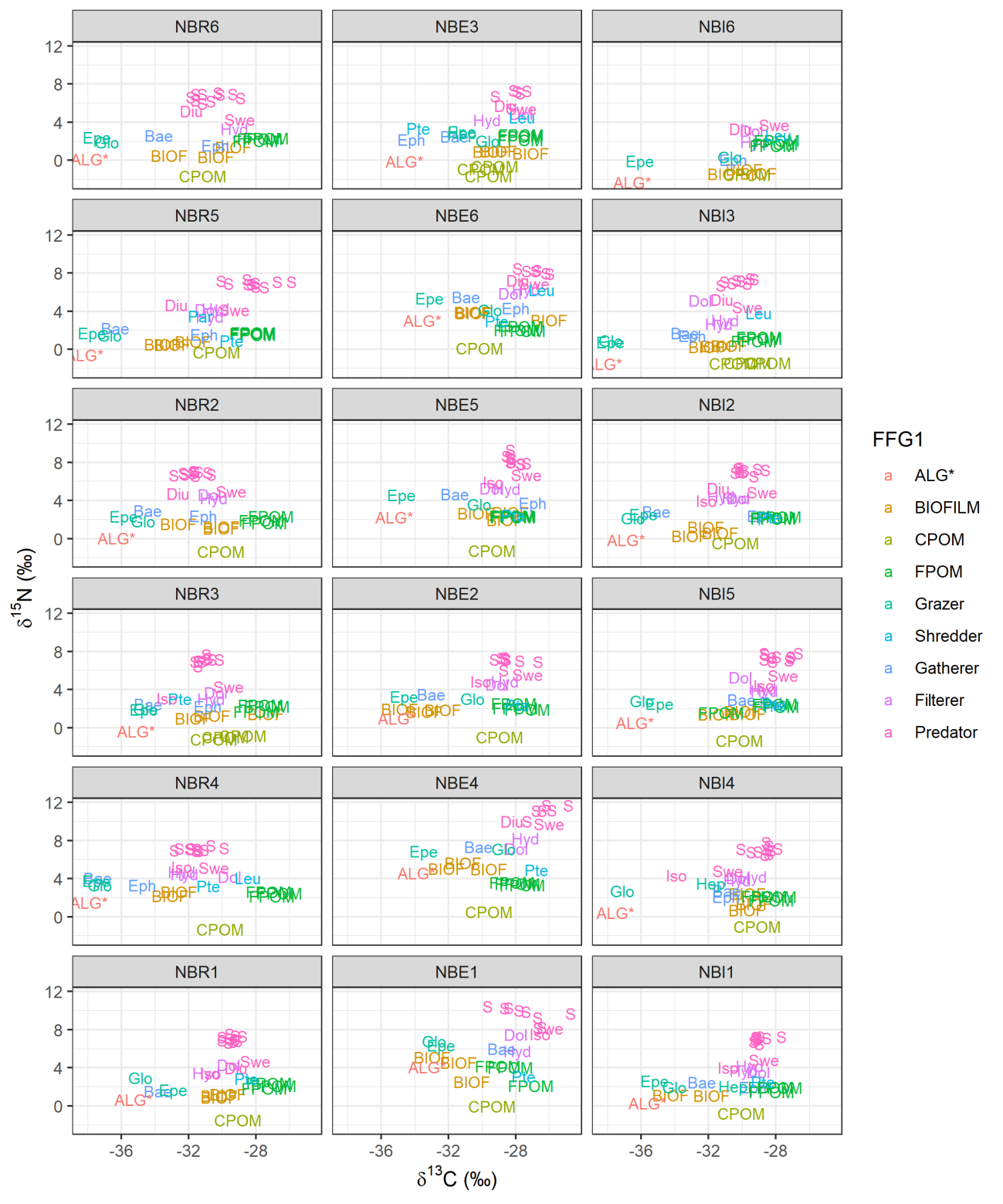


**Fig. S2:** Carbon (δ^13^C, ‰) and nitrogen (δ^15^N, ‰) stable isotope biplots for 6 stream-sites (sorted from smallest to largest) within three basins ranging in forest management intensity (intensive – NBI, extensive – NBE, minimal – NBR). Consumers (benthic macroinvertebrates [Bae – Baetis, Eph – Ephemerella, Glo – Glossosoma, Hep – Heptageniidae, Hyd – Hydropsychidae, Dol – Dolophilodes, Swe – Sweltsa, Per – Perlodidae, Leu – Leuctra, Pte – Pteronarcys] and sculpin [S]) are shown in relation to potential food sources (CPOM, FPOM, biofilm and algae [calculated]).


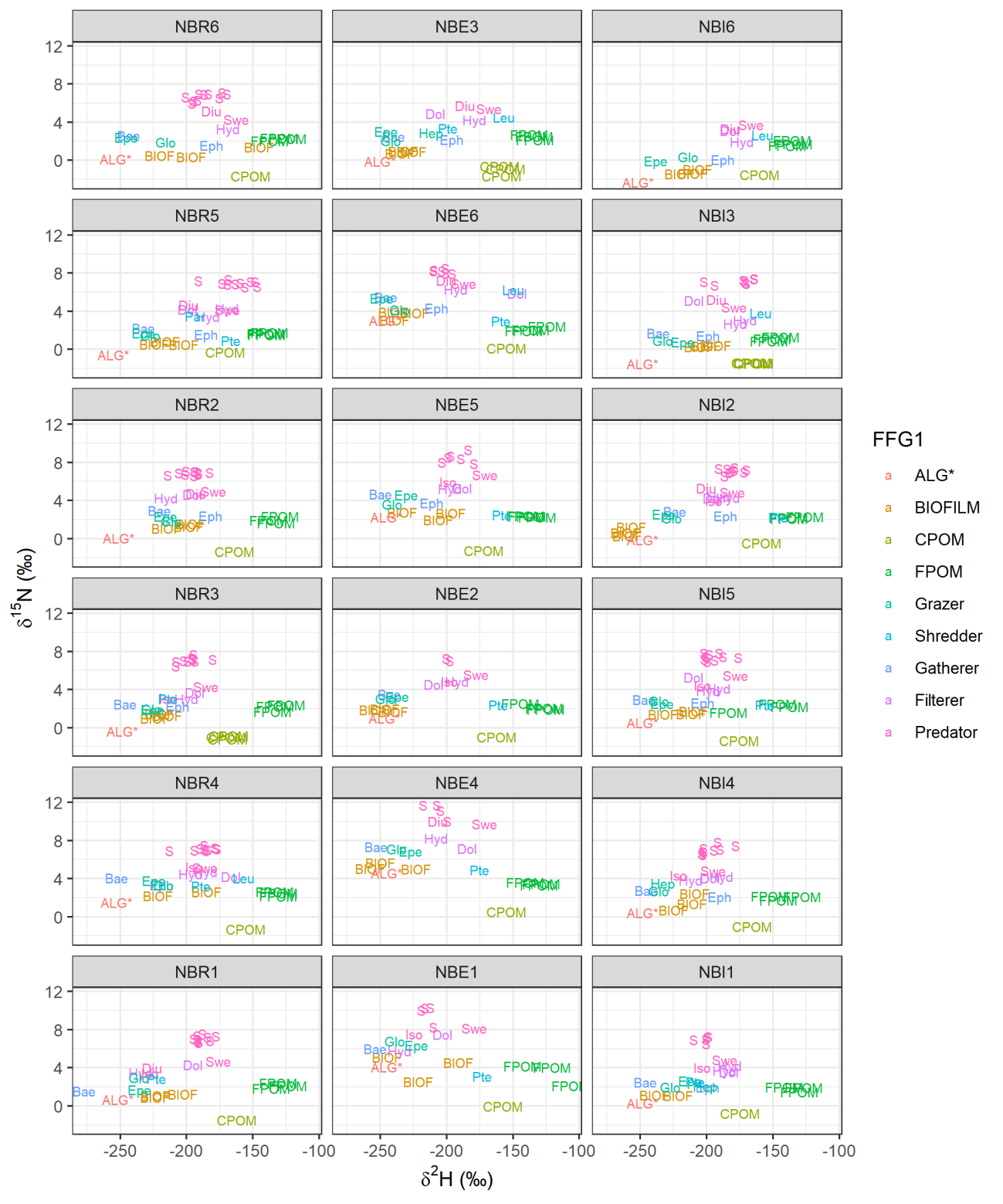


**Fig. S3:** Hydrogen (δ^2^H, ‰) and nitrogen (δ^15^N, ‰) stable isotope biplots for 6 stream-sites (sorted from smallest to largest) within three basins ranging in forest management intensity (intensive – NBI, extensive – NBE, minimal – NBR). Consumers (benthic macroinvertebrates [Bae – Baetis, Eph – Ephemerella, Glo – Glossosoma, Hep – Heptageniidae, Hyd – Hydropsychidae, Dol – Dolophilodes, Swe – Sweltsa, Per – Perlodidae, Leu – Leuctra, Pte – Pteronarcys] and sculpin [S]) are shown in relation to potential food sources (CPOM, FPOM, biofilm and algae [calculated]).


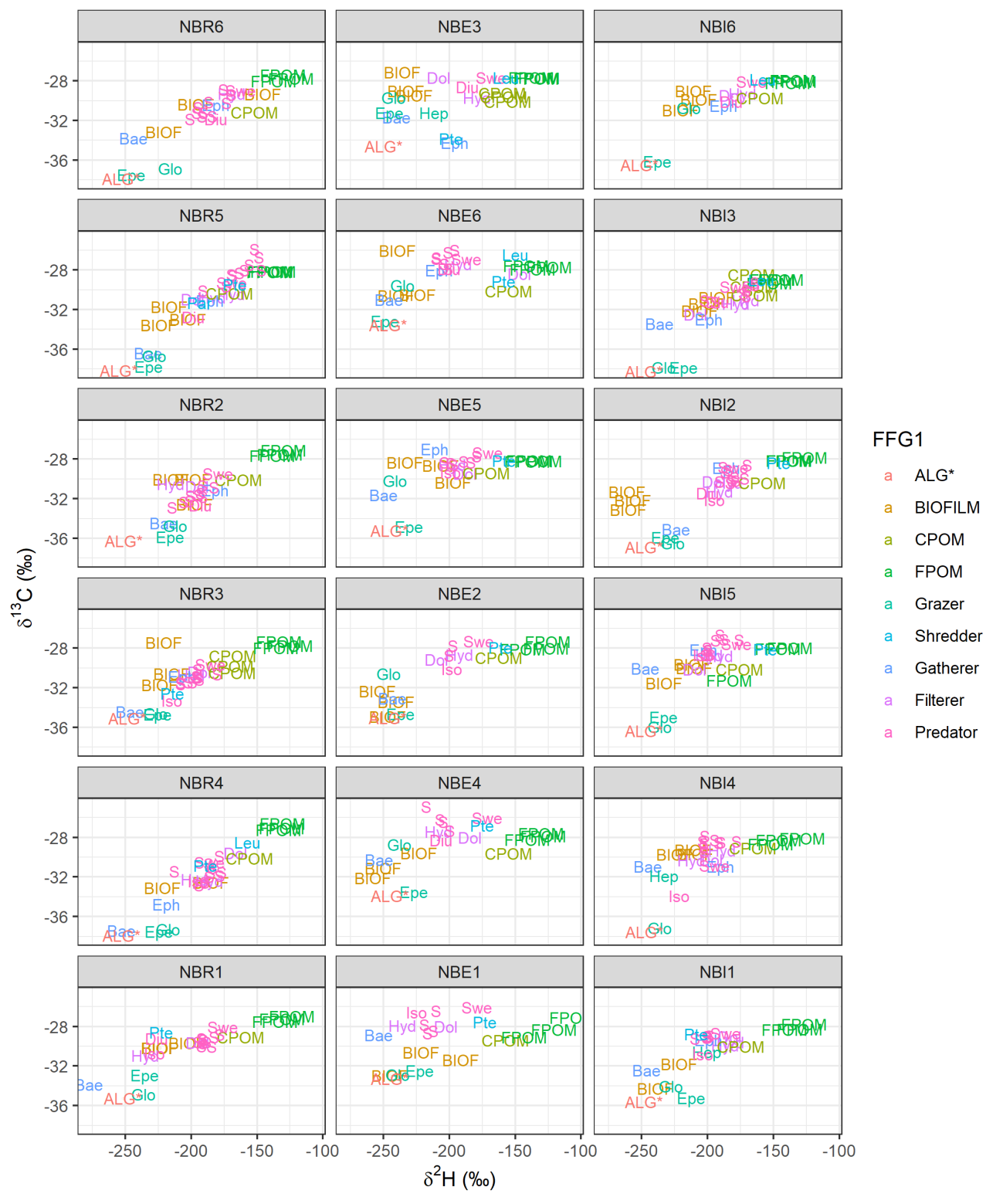


**Fig. S4:** Carbon (δ^13^C, ‰) and hydrogen (δ^2^H, ‰) stable isotope biplots for 6 stream-sites (sorted from smallest to largest) within three basins ranging in forest management intensity (intensive – NBI, extensive – NBE, minimal – NBR). Consumers (benthic macroinvertebrates [Bae – Baetis, Eph – Ephemerella, Glo – Glossosoma, Hep – Heptageniidae, Hyd – Hydropsychidae, Dol – Dolophilodes, Swe – Sweltsa, Per – Perlodidae, Leu – Leuctra, Pte – Pteronarcys] and sculpin [S]) are shown in relation to potential food sources (CPOM, FPOM, biofilm and algae [calculated]).

# Mixing model results

**Table S2:** Mean (range) proportion of algal contribution to different invertebrate taxa and sculpin (rows) calculated based on C-H mixing models in three basins ranging in forest management intensity.

| Taxon | Intensive | Extensive | Minimal | All basins |
| --- | --- | --- | --- | --- |
| Heptageniidae | 0.89 (0.83-0.91) | 0.92 (0.85-0.96) | 0.92 (0.87-0.96) | 0.91 (0.83-0.96) |
| *Glossosoma* | 0.88 (0.73-0.92) | 0.90 (0.80-0.97) | 0.90 (0.83-0.96) | 0.89 (0.73-0.97) |
| *Baetis* | 0.83 (0.72-0.93) | 0.87 (0.77-0.96) | 0.87 (0.77-0.95) | 0.86 (0.72-0.96) |
| *Ephemerella* | 0.62 (0.49-0.77) | 0.85 (0.74-0.96) | 0.66 (0.35-0.95) | 0.71 (0.35-0.96) |
| *Dolophilodes* | 0.61 (0.46-0.80) | 0.73 (0.38-0.95) | 0.61 (0.25-0.88) | 0.65 (0.25-0.95) |
| Hydropsychidae | 0.48 (0.32-0.74) | 0.76 (0.59-0.97) | 0.65 (0.32-0.95) | 0.63 (0.32-0.97) |
| *Pteronarcys* | 0.39 (0.11-0.82) | 0.61 (0.22-0.92) | 0.62 (0.14-0.95) | 0.56 (0.11-0.95) |
| *Leuctra* | 0.15 (0.14-0.16) | 0.49 (0.35-0.58) | 0.29 (0.15-0.43) | 0.31 (0.14-0.58) |
| Perlodidae | 0.82 (0.69-0.89) | 0.88 (0.81-0.93) | 0.80 (0.69-0.90) | 0.83 (0.69-0.93) |
| *Sweltsa* | 0.79 (0.53-0.89) | 0.87 (0.76-0.95) | 0.69 (0.41-0.90) | 0.78 (0.41-0.95) |
| Sculpin | 0.14 (0.03-0.30) | 0.56 (0.07-0.91) | 0.47 (0.09-0.87) | 0.40 (0.03-0.91) |
| All taxa | 0.64 (0.53-0.71) | 0.78 (0.61-0.92) | 0.70 (0.50-0.88) | 0.71 (0.50-0.92) |


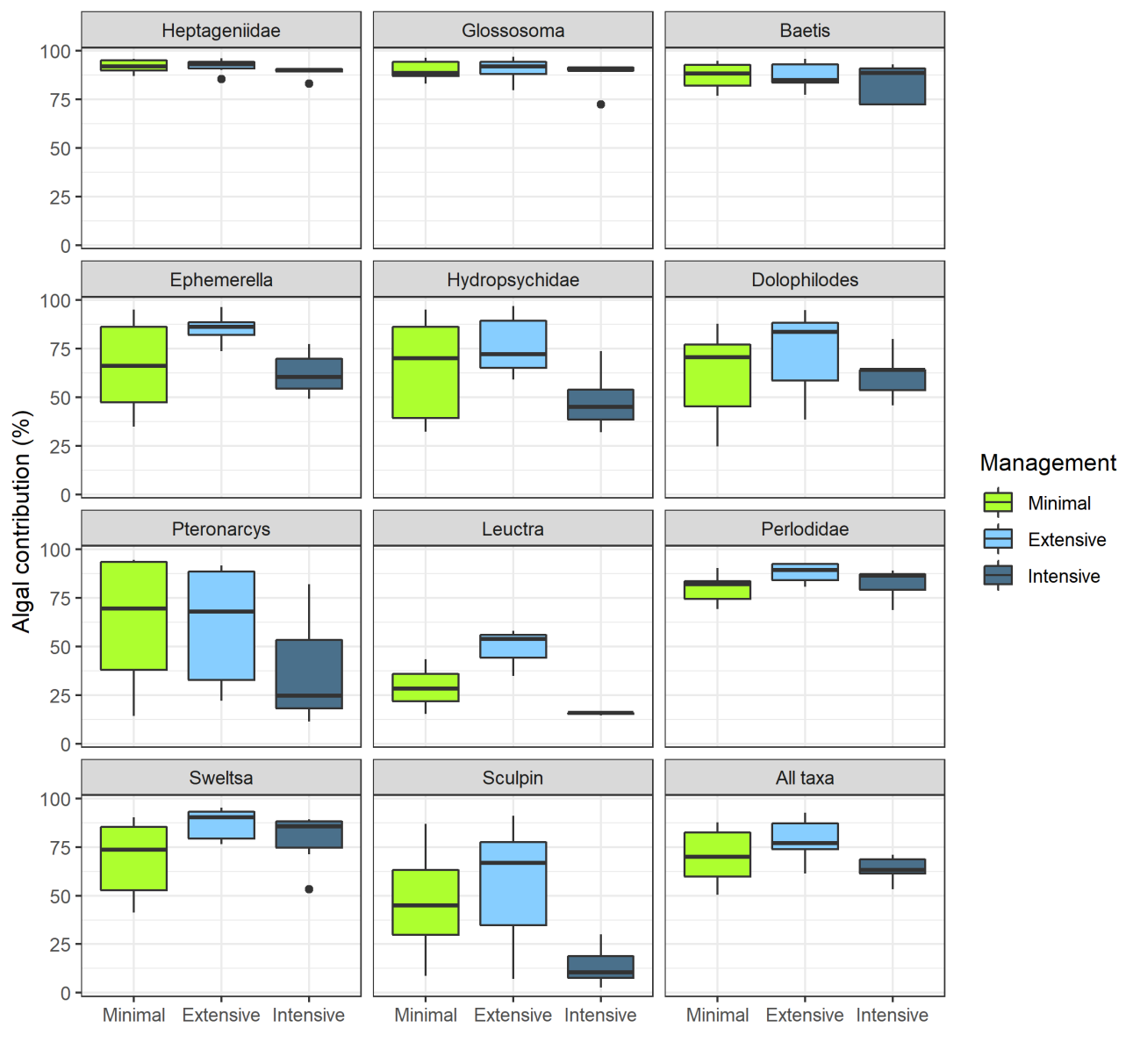


**Fig. S5:** Boxplots showing the % autochthony in different macroinvertebrate taxa and sculpin calculated based on C-H mixing models in three basins ranging in forest management intensity.

# Regression results

**Table S3:** Results of linear regressions between δ^2^H in terrestrial food sources and stream consumers (rows) and drainage area in three basins ranging in forest management type (columns). The table shows the *F-* and *p*-values from ANOVAs testing the significance of the interaction term (i.e., different slopes for the terrestrial food source and consumers); *p* ≤ 0.10 are bolded.

| δ^2^H | Intensive | | Extensive | | Minimal | |
| --- | --- | --- | --- | --- | --- | --- |
|  | *F* | *p* | *F* | *p* | *F* | *p* |
| *Baetis* | 2.4 | 0.17 | 1.4 | 0.27 | 3.1 | 0.11 |
| *Ephemerella* | 0.005 | 0.94 | 0.09 | 0.77 | 6.3 | **0.04** |
| Heptageniidae | 2.7 | 0.14 | 8.9 | **0.02** | 0.5 | 0.51 |
| *Glossosoma* | 0.7 | 0.42 | 0.01 | 0.92 | 1.1 | 0.33 |
| Hydropsychidae | 1.5 | 0.26 | 7.3 | **0.03** | 7.2 | **0.03** |
| *Dolophilodes* | 0.01 | 0.92 | 0.3 | 0.61 | 0.4 | 0.54 |
| *Pteronarcys* | 16.8 | **0.009** | 0.9 | 0.37 | 4.8 | **0.07** |
| *Sweltsa* | 3.4 | **0.10** | 0.01 | 0.92 | 1.2 | 0.30 |
| Perlodidae | 4.6 | **0.06** | 6.5 | **0.03** | 6.4 | **0.03** |
| Sculpin | 15 | **0.006** | 1.4 | 0.27 | 0.1 | 0.70 |

**Table S4:** Results of linear regressions between percent algal contribution to invertebrate and sculpin taxa and clearcut intensity (2008-2017) in three basins ranging in forest management type. The table shows the *p*-values from ANOVAs testing the significance of each fixed variable in within basin and within taxon models; *p* ≤ 0.10 are bolded.

| Within basin ^1^ | | | |
| --- | --- | --- | --- |
|  | Clearcut | Taxon | Clearcut*Taxon |
| Intensive | **0.001** | **<0.001** | 0.54 |
| Extensive | 0.49 | **0.002** | 0.55 |
| Minimal | **<0.001** | **<0.001** | **0.001** |
| Within taxa ^2^ | | | |
|  | Clearcut | Type | Clearcut*Type |
| *Baetis* | 0.49 | 0.61 | 0.67 |
| Heptageniidae | **0.10** | **0.07** | 0.37 |
| *Glossosoma* | 0.23 | 0.27 | **0.002** |
| *Ephemerella* | 0.23 | **0.01** | **0.03** |
| Hydropsychidae | 0.64 | **0.03** | **0.002** |
| *Dolophilodes* | **0.02** | **0.05** | **0.05** |
| *Pteronarcys* | 0.19 | 0.31 | 0.18 |
| *Leuctra* | 0.18 | 0.21 | 0.91 |
| Perlodidae | 0.32 | **0.09** | **0.008** |
| *Sweltsa* | 0.66 | **0.07** | **0.01** |
| Sculpin | 0.75 | **0.08** | 0.17 |

^1^ Within basin models: Algal contribution = Clearcut x Taxon

^2^ Within taxa models: Algal contribution = Clearcut x Basin type

**
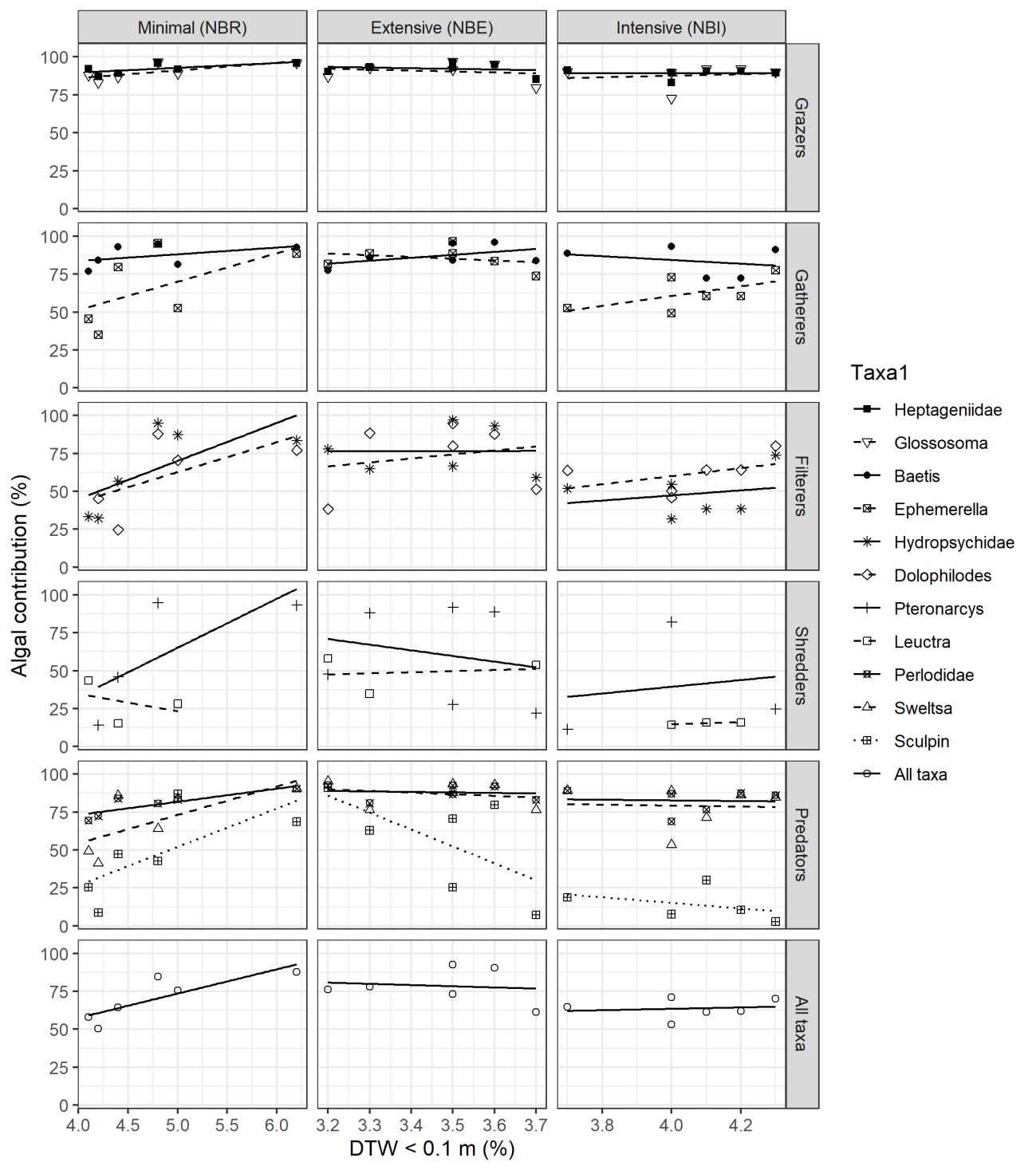
**

**Fig. S6:** Linear relationship between % autochthony (y-axis) in 8 invertebrate taxa and sculpin classified according to their functional feeding group (rows) and % catchment with depth-to-water (DTW) values lower than 0.10 m (x-axis) in three basins differing in forest management intensity (columns). Six sites per basin were sampled.

**Table S5:** Results of linear regressions between the range in δ^15^N between sculpin and macroinvertebrate primary consumers and catchment variables (rows) in three basins ranging in forest management type (intensive – NBI, extensive – NBE, minimal – NBR). The table shows the *p*-values from ANOVAs testing the significance of each explanatory variable (EV) and EV x Basin type interaction (INT), as well as the *p-*values and sign of the slope (+/-) from regression models within each basin (NBI, NBE, NBR); *p* ≤ 0.10 are bolded.

|  | EV | INT | NBI | NBE | NBR |
| --- | --- | --- | --- | --- | --- |
| Drainage area | 0.21 | 0.56 | 0.21 (-) | 0.41 (-) | 0.95 (+) |
| Crossing density | 0.11 | 0.55 | 0.41 (-) | **0.03 (-)** | 0.71 (+) |
| Road density | 0.77 | **0.09** | **0.07 (-)** | 0.32 (+) | 0.56 (+) |
| Clearcut | 0.21 | 0.57 | 0.47 (+) | 0.13 (-) | 0.59 (-) |
| Total disturbance | 0.33 | 0.40 | 0.95 (-) | **0.06 (-)** | 0.41 (+) |
| Slope | 0.65 | 0.38 | 0.26 (-) | 0.77 (+) | 0.65 (-) |
| DTW < 0.1m | 0.48 | 0.96 | 0.95 (-) | 0.73 (+) | 0.52 (+) |
| Forest height | 0.76 | 0.50 | 0.81 (+) | 0.42 (+) | 0.38 (-) |
| Deciduous cover | 0.25 | 0.21 | **0.09 (+)** | 0.96 (+) | 0.41 (+) |
